# SerpinA3 in the Early Recognition of Acute Kidney Injury to Chronic Kidney Disease (CKD) transition in the rat and its Potentiality in the Recognition of Patients with CKD

**DOI:** 10.1038/s41598-019-46601-1

**Published:** 2019-07-17

**Authors:** Andrea Sánchez-Navarro, Juan M. Mejía-Vilet, Rosalba Pérez-Villalva, Diego L. Carrillo-Pérez, Brenda Marquina-Castillo, Gerardo Gamba, Norma A. Bobadilla

**Affiliations:** 1 Molecular Physiology Unit, Instituto de Investigaciones Biomédicas, Universidad Nacional Autónoma de México, Mexico City, Mexico; 2 Department of Nephrology and Mineral Metabolism, Instituto Nacional de Ciencias Médicas y Nutrición Salvador Zubirán, Mexico City, Mexico; 3 Department of Experimental Pathology, Instituto Nacional de Ciencias Médicas y Nutrición Salvador Zubirán, Mexico City, Mexico; 4 Deparment of Internal Medicine, Instituto Nacional de Ciencias Médicas y Nutrición Salvador Zubirán, Mexico City, Mexico; 5 Tecnológico de Monterrey, Escuela de Medicina y Ciencias de la Salud, Monterrey, Nuevo León, Mexico

**Keywords:** Kidney, Chronic kidney disease

## Abstract

Recognizing patients at early phases of chronic kidney disease (CKD) is difficult, and it is even more challenging to predict acute kidney injury (AKI) and its transition to CKD. The gold standard to timely identify renal fibrosis is the kidney biopsy, an invasive procedure not usually performed for this purpose in clinical practice. SerpinA3 was identified by high-resolution-mass-spectrometry in urines from animals with CKD. An early and progressive elevation of urinary SerpinA3 (uSerpinA3) was observed during the AKI to CKD transition together with SerpinA3 relocation from the cytoplasm to the apical tubular membrane in the rat kidney. uSerpinA3/alpha-1-antichymotrypsin was significantly increased in patients with CKD secondary to focal and segmental glomerulosclerosis (FSGS), ANCA associated vasculitis (AAV) and proliferative class III and IV lupus nephritis (LN). uSerpinA3 levels were independently and positively associated with renal fibrosis. In patients with class V LN, uSerpinA3 levels were not different from healthy volunteers. uSerpinA3 was not found in patients with systemic inflammatory diseases without renal dysfunction. Our observations suggest that uSerpinA3 can detect renal fibrosis and inflammation, with a particular potential for the early detection of AKI to CKD transition and for the differentiation among lupus nephritis classes III/IV and V.

## Introduction

Chronic kidney disease (CKD) is a silent disease that is often not recognized in clinical practice until the global renal function is impaired or proteinuria is detected in urine assays. Over the past two decades, the incidence of CKD has increased more than threefold, and according to the World Health Organization, it will be one of the three main causes of death and disability in the world by 2020^1^. To assess renal function, international organizations such as the National Kidney Foundation in the Kidney Disease Outcomes Quality Initiative (NKF-KDOQI) or the Kidney Disease Improving Global Outcomes (KDIGO) have proposed a series of guidelines for CKD detection that recommend CKD screening with serum creatinine, urea nitrogen and the abnormal presence of proteins in the urine^2,3^. Unfortunately, these classical kidney injury markers are only detectable when the disease is already advanced, and pharmacological treatments are possibly less effective. Moreover, tubulointerstitial fibrosis has been shown to occur earlier than renal dysfunction, and when proteinuria appears, the disease has been already established^4,5^. The presence of renal fibrosis, before glomerular filtration rate (GFR) reduction, can only be documented by kidney biopsy. Additionally, for most glomerular diseases, as is the case for lupus nephritis (LN), kidney biopsy represents the gold standard for diagnosis and the only way to classify histological damage for diagnostic and prognostic purposes^5^.

Acute kidney injury (AKI) represents another important renal disease around the globe. It affects 21% of hospitalized patients in general wards and up to 60% of patients in critical care units^6,7^. It was previously speculated that patients who completely recovered from an AKI episode had no further repercussions on kidney function and structure; however, recent evidence based on epidemiological and experimental observations has demonstrated that in many cases, AKI leads to CKD^8–13^. Therefore, AKI is now acknowledged as a risk factor for the development of CKD and an accelerating factor for the transition from CKD to end-stage renal disease (ESRD)^13–15^.

Currently, CKD diagnosis is made through urine proteinuria or albuminuria, serum creatinine elevation, urine sediment abnormalities, imaging studies or histopathology. The disadvantage to these approaches is the late detection of renal disease and, for the case of histopathological studies, the invasiveness and impracticability. Therefore, current efforts are under way to identify a timely non-invasive biomarker for CKD and the AKI to CKD transition.

We identified the abnormal presence of serpinA3 in urine samples from animals with CKD by high-resolution mass spectrometry, and here, we present evidence that uSerpinA3 is a potentially useful diagnostic marker to detect the AKI to CKD transition and CKD from different etiologies.

## Methods

All experiments involving animals were conducted in accordance with the NIH Guide for the Care and Use of Laboratory Animals (https://grants.nih.gov/grants/olaw/guide-for-the-care-and-use-of-laboratory-animals.pdf) and with the Mexican Federal Regulation for animal reproduction, care, and experimentation (NOM-062-ZOO-2001). All methods involving humans were performed in accordance with the relevant guidelines and regulations. The study was approved by both the Animal Care and Use Committee and the Ethical Committee for human research at Instituto Nacional de Ciencias Médicas y Nutrición Salvador Zubirán. Informed consent was obtained from all the patients included in this study.

All the animals used in this study were maintained in controlled conditions of temperature and humidity in our animal housing facility with 12:12 h day/night cycle, with free access to water and food.

### AKI to CKD transition model

36 male Wistar rats (320–350 g) were included and randomly distributed into two groups as follows: control animals who underwent sham surgery and were studied and sacrificed at 1, 2, 3, or 4-months (n = 4 per period), and rats who underwent right nephrectomy and unilateral 45-min left renal ischemia who were studied and sacrificed at 1, 2, 3, or 4-months (n = 5 per period). In our experience, 30–40% of the animals died after 24 h of ischemic injury, but none was lost during the follow-up (4 months). The animals were maintained in controlled conditions in our animal facility. All the samples of the included animals were evaluated and included in the analyses.

### Ischemia/reperfusion model

After an intra-peritoneal injection of sodium pentobarbital (30 mg/kg), the rats were placed on a heating pad to maintain core body temperature at 37 °C. Unilateral renal ischemia was induced using a non-traumatic clamp on left renal artery for 45 min. Then, the clip was released to allow the return of oxygenated blood to the kidney and right nephrectomy was performed. 3–0 vicryl and silk sutures were used to close the muscle and the skin, respectively. For sham surgery, laparotomy and renal pedicle dissection, without clamping, was performed.

### Functional studies

At the end of the experimental period, rats were anesthetized with sodium pentobarbital (30 mg/kg) and placed on a homoeothermic table. The femoral arteries were catheterized with polyethylene tubing (PE-50). The mean arterial pressure (MAP) was monitored with a pressure transducer (model p23 db, Gould) and recorded on a polygraph (Grass Instruments, Quincy, MA). An ultrasound transit-time flow probe (transonic flow probe, New York, NY) was placed around the left artery and filled with ultrasonic coupling gel (HR Lubricating Jelly, Carter-Wallace, New York, NY) to record the renal blood flow (Transonic flowmeter, New York, NY). Blood samples were taken at the end of the study as we previously reported^16,17^.

### Biochemical studies

Turbidimetric method with trichloroacetic acid (TCA) was employed to measure urinary protein excretion monthly in 24-h urine collections throughout the follow-up in all studied groups. Quantichrom creatinine assay kit (DICT-500) was used to determine urine and serum creatinine concentrations and renal creatinine clearance was obtained using the standard formula.

### Percentage of tubulo-interstitial fibrosis

Paraffin embedded renal tissue was deparaffinized and 3 µm sections were stained with Sirius red. Eight to ten subcortical fields (magnification x400) were recorded from each kidney slide using a digital camera incorporated in a Nikon Light microscope to measure the degree of tubulo-interstitial fibrosis by morphometry. The affected area of tubulo-interstitial fibrosis was automatically quantified by an eclipse net software. All the analyses were performed by an observer blind to the experiments as we previously reported^16,17^.

### High-resolution urine mass spectrometry

Bands of 55 and 70 kDa were cut from an 8.5% acrylamide gel stained with Coomassie R-250 blue to perform a tryptic protein digestion in gel, following the protocol of the Laboratory of Proteomics from our University. The collected peptides were desalted with Zip Tip C18 millipore tips (LUP protocol) and dried in a speedvac. Samples were kept at −80 °C until analysis. The sample ionization and subsequent analysis was performed by high-resolution mass spectrometry (LTQ Orbitrap, Thermo Fisher Scientific).

### Renal SerpinA3 mRNA levels

At every predefined period, animals were sacrificed and half of the left kidney was quickly removed and frozen for molecular studies. Total RNA was isolated from the kidney using the TRIzol method (Invitrogen, Carlsbad, CA) and its integrity checked integrity using 1% agarose gel electrophoresis. To avoid DNA contamination, total RNA samples were treated with DNAase (DNAase I; Invitrogen). Reverse transcription (RT) was carried out with 1 μg of total RNA and 200 U of Moloney’s murine leukemia virus reverse transcriptase (Invitrogen) as we previously reported^16,17^. SerpinA3 mRNA levels (Rn04280570_m1) were quantified by real-time PCR on an ABI Prism 7300 Sequence Detection System (TaqMan, ABI, Foster City, CA). Eukaryotic 18S rRNA (predesigned assay reagent Applied by ABI, external run, Rn03928990_g1) was used as endogenous control. The relative quantification of each gene expression was performed with the comparative threshold cycle (Ct) method.

### SerpinA3 protein levels in tissue, urine and plasma

Electrophoresis on an 8.5% denaturing acrylamide gel was performed with 40 μg of renal tissue proteins, 1 μl of rat urine, 15 μl of human urine or 30 nl of human plasma as appropriate. Proteins were then transferred to pre-equilibrated polyvinyl difluoride (PVDF, Millipore) membranes with 1x transfer buffer (190 mM glycine, 2 mM Tris base, 0.1% SDS) in a transblot (SD cell, BioRad) for 60 min at 9 volts. Subsequently, membranes were blocked for 90 min with TBS buffer 5% blocking agent (BioRad). After blockade, membranes were incubated with the primary antibody serpinA3K (1:2000, Santa Cruz, SC-162175 for rat samples) and antibody serpinA3/alpha-1-antichymotrypsin (Proteintech, 55480-1-AP for human samples) overnight at 4 °C. The membranes were then incubated with the HRP-coupled anti-goat secondary antibody or HRP-coupled anti-rabbit secondary antibody, respectively (1:10000, Millipore or Thermo Scientific Pierce, respectively).

### Urine Hsp72 levels

Urine Hsp72 levels were detected by Western blot. Each urine sample was diluted 1:10 in 0.9% saline solution, and 10 μL were loaded and resolved by 8.5% SDS-PAGE electrophoresis and then electroblotted. The membranes were incubated with mouse anti-Hsp72 antibody (ENZO Life Sciences, 1:5000 dilution) for 2-h. Thereafter; membranes were incubated with a secondary antibody, HRP-conjugated goat anti-mouse IgG (1:5000, Santa Cruz Biotechnology). The proteins were detected using a commercial chemiluminiscence kit (Millipore), as we previously described^18^.

### CKD patient sample collection

Plasma and urine samples were collected pre-biopsy from male and female patients diagnosed with focal and segmental glomerulosclerosis (FSGS, n = 14), lupus nephritis (LN, n = 47), and ANCA associated vasculitis (AAV, n = 19). Lupus nephritis biopsies were classified according to the ISN/RPS classification^19^ into class III (n = 18), class IV (n = 18) and class V (n = 11). The sample size was based on the availability of biological samples and not statistically determined. The percentage of tubulointerstitial fibrosis was evaluated in renal biopsies by an expert nephropathologist. Patients with inflammatory diseases but without renal dysfunction were also included: liver cirrhosis (LC, n = 6), acute pancreatitis (AP, n = 6) and active rheumatoid arthritis (RA, n = 6). All the groups were compared to healthy volunteers (n = 20).

*Acute pancreatitis w*as defined by the presence of two of the following: acute onset of persistent, severe, epigastric pain often radiating to the back, elevation in serum lipase or amylase to three times or greater than the upper limit of normal, or characteristic findings in the image studies^20^. RA was diagnosed by the 2010 ACR/EULAR classification criteria. Active RA was defined as Disease Activity Score-28 joints (DAS28)-erythrocyte sedimentation rate (ESR) ≥ 3.2^21^. LC was diagnosed by liver biopsy and/or clinical, biochemical, ultrasound, and/or endoscopic findings, and classified according to the patient’s Child-Pugh score^22^.

### SerpinA3 Immunohistochemistry

Tissues from rat or human biopsies embedded in paraffin and cut into 4 μm sections in charged slides. Once deparaffinized, the antibody recovery was performed with citrate buffer (Bio SB) for 12 min in high pressure. The slides were then blocked with a inmunoDNA background blocker (Bio SB) for 20 min and incubated with the primary serpinA3 antibody (1:500, Santa Cruz, SC-162175) for 2 h at 23 °C. Then the slides were incubated with a goat-on-rodent-HRP-polymer-secondary antibody (Biocare medical), and revealed with DAB peroxidase Substrate (Bio SB).

### uSerpinA3 levels by ELISA

Human serpinA3/Alpha-1-antichymotrypsin concentration in the urine samples was analyzed by ELISA kit (Catalog No. ELH-serpinA3, Raybiotech Inc.) The procedure was performed following the instructions of the manufacturer. Urine samples from healthy volunteers and patients were diluted at 1:100 or 1:200–500, respectively.

### Statistical analysis

Data distribution was evaluated by the Shapiro-Wilk test. The variables with normal distributions are presented as mean ± SD, while variables with non-normal distributions are presented as median and interquartile range. The groups were compared with ANOVA or the Kruskal Wallis test as appropriate, with Bonferroni’s and Dunn’s tests for multiple comparisons. Correlations were evaluated by Spearman’s test. The factors independently associated with interstitial fibrosis in LN patients were determined by a linear regression model. Variables were log-transformed and collinearity assessed by the variation inflation factor (VIF). Statistical significance was defined as a p-value < 0.05.

## Results

### Identification of SerpinA3 in the urine from rats with CKD

**Figure 1:**
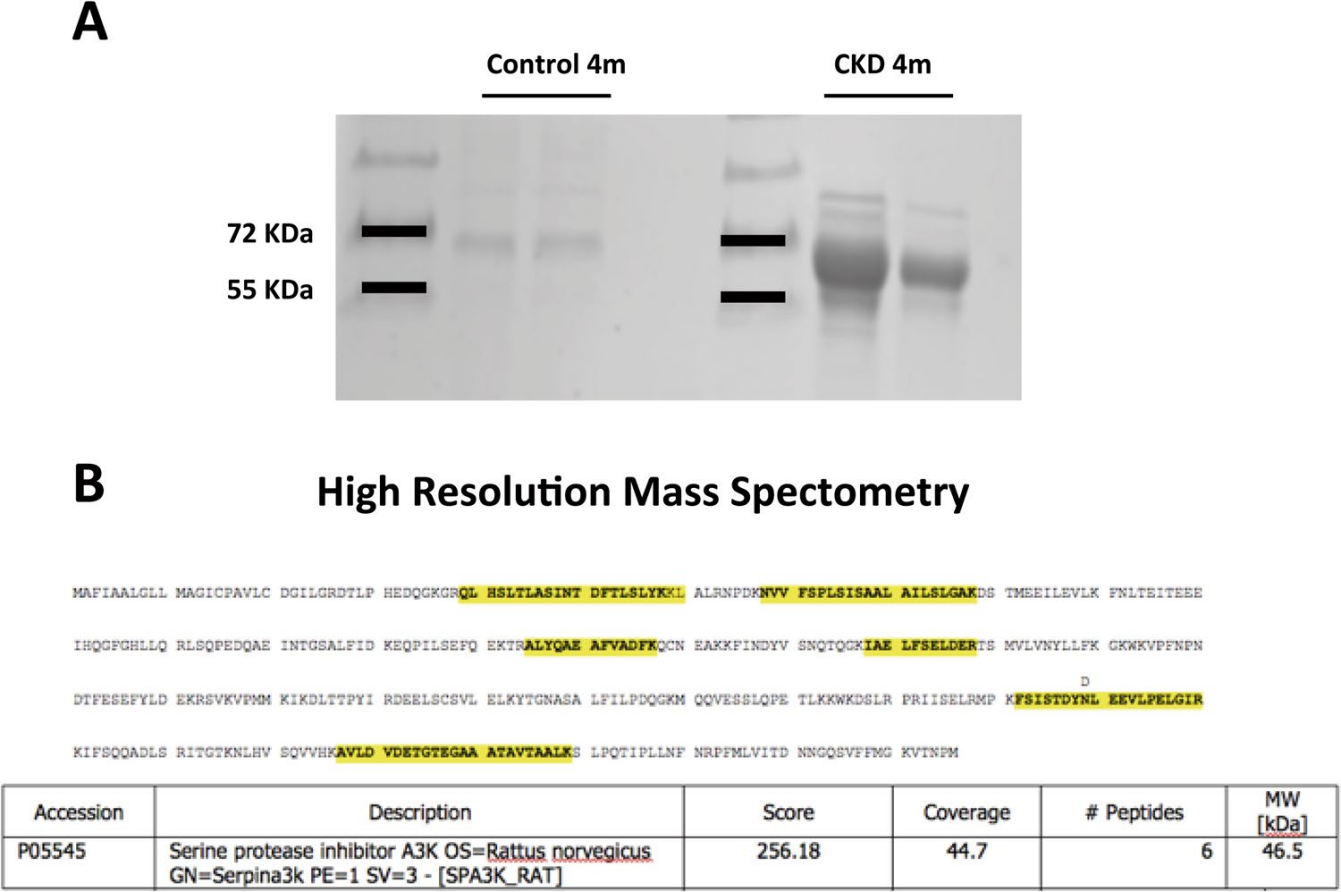
Urine high-resolution mass spectrometry in CKD rats. (**A**) Representative acrylamide gel under denaturing conditions and subsequent staining with Coomassie blue of urines from rats with CKD compared to control rats (sham). The identified proteins were mainly between 55 and 72 KDa. (**B**) SerpinA3 was identified with high-resolution mass spectrometry with a high coverage (44.7%) in urine samples from CKD rats.

CKD was induced in male rats using the model of right nephrectomy plus contralateral renal ischemia for 45-min then followed for four months. By the end of the study, all animals developed CKD and exhibited higher amounts of urine proteins between 55 and 72 kDa compared to the control group (Fig. 1A). The bands were isolated, and the proteins were extracted from the gel to be analyzed by high-resolution mass spectrometry. As expected, albuminuria was present in the urine. In addition, serpinA3 was identified in these samples with a coverage of 44.7%. Other identified proteins were serotransferrin, alpha-1-antiproteinase, serine A3M protease inhibitor, LOC299282 protein, serpinC1, kininogen T, B-fetuin, and type I keratin. However, albumin and serpinA3 constituted the most abundant proteins (Fig. 1B).

### uSerpinA3 levels in the AKI to CKD transition

**Figure 2:**
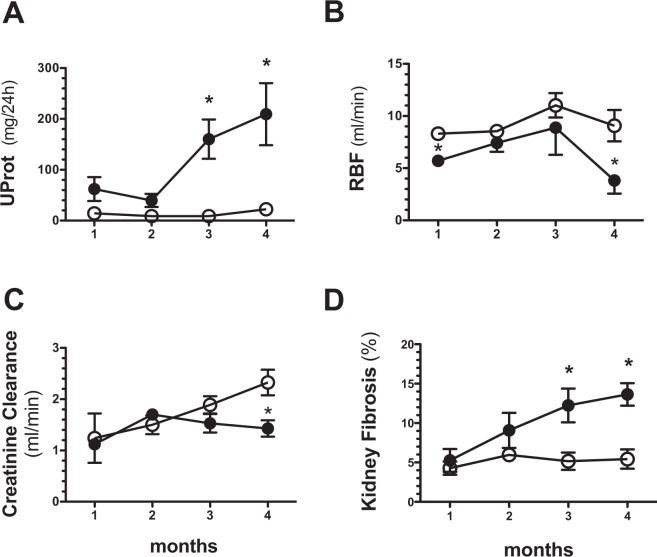
Temporal course of renal dysfunction and fibrosis in AKI to CKD transition in the rat. (**A**) Proteinuria, (**B**) Renal blood flow (RBF), (**C**) Creatinine clearance, and (**D**) Tubulo-interstitial fibrosis since the 1^st^ until the 4^th^ month post-ischemia. Data are represented as the mean ± SE (for sham, n = 4, and for the AKI to CKD transition groups, n = 5 per period). White circles represent sham and black circles represent AKI to CKD transition groups. *p < 0.05 vs. sham group in their respective period.

We analyzed the time course of the AKI to CKD transition by studying and euthanizing groups of animals on a monthly basis, starting in the 1^st^ month and continuing up to the 4^th^ month post-ischemia. The AKI to CKD transition was characterized by a progressive elevation of proteinuria that was statistically significant starting at the 3^rd^ month (Fig. 2A), together with a significant reduction of renal blood flow (Fig. 2B) and renal dysfunction at the 4^th^ month (Fig. 2C). The development of tubulointerstitial fibrosis was also observed starting at the 3^rd^ month post-ischemia (Fig. 2D). These results show that uninephrectomized rats exposed to an AKI episode developed progressive CKD that was detected between three- and four-months post-AKI by the classic renal injury biomarkers.

**Figure 3:**
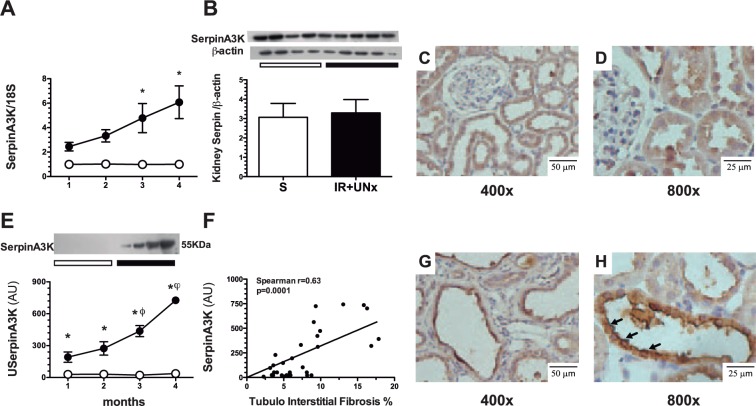
Timely AKI to CKD transition detection by serpinA3. (**A**) SerpinA3 mRNA levels in renal cortex during the follow-up of the animals, (**B**) SerpinA3 protein levels in the renal cortex evaluated by Western Blot 4 months post-ischemia, (**C**,**D**) representative microphotographs of serpinA3 immunostaining in renal cortex from the control group, magnification 400 and 800x respectively, (**E**) Urinary serpinA3 levels during AKI to CKD transition and compared with the control group, (**F**) Spearman correlation between urine serpinA3 and tubulo-interstitial fibrosis, and (**G**,**H**) representative microphotographs of serpinA3 immunostaining in renal cortex from the AKI to CKD transition group, magnification 400 and 800x respectively. Data are represented as mean ± SE. (for sham, n = 4, and for the AKI to CKD transition groups, n = 5 per period). White circles or bar represent sham and black circles or bar represent AKI to CKD transition groups and. *p < 0.05 vs. the sham group in their respective period, ^ϕ^p < 0.05 vs. IR + UNx 2^nd^ month and ωp < 0.05 vs. IR + UNx 3^rd^ month.

Renal tissue serpinA3 mRNA and protein levels, as well as uSerpinA3 were analyzed during the time course of the AKI to CKD transition, as shown in Fig. 3. We observed a significant increase in serpinA3 mRNA levels starting at the 3^rd^ month post-ischemia compared to the control group (Fig. 3A); however, this difference was not reflected at the protein level, as is shown by the Western blot analysis, in which no differences between groups were observed (Fig. 3B). Immunohistochemical analysis revealed that in the control group, serpinA3 is expressed mostly in the cytoplasm of tubular epithelium (Fig. 3C,D). Interestingly, in the AKI to CKD transition group serpinA3 is relocated to the apical membrane (Fig. 3G,H). The Supp. Fig. 1 shows the serpinA3 relocation along the AKI to CKD transition, that was slightly observed since the 1^st^ month and the relocation was even more evident throughout the CKD progression. Moreover, we found that the AKI to CKD transition was timely revealed by a progressive increase in uSerpinA3 levels, which were significantly elevated starting at the 1^st^ month, even before the proteinuria appearance (Fig. 2A). In contrast, this protein was not detected in the urine from the control group (Fig. 3E). It is noteworthy that 15 days after the AKI episode was resolved, serpinA3 was not found in the urine (Supp. Fig. 2). Thus, the abnormal uSerpinA3 levels and the gradual increment along the time course reflect the AKI to CKD transition. Accordingly to these findings, there was a significant correlation between renal fibrosis and uSerpinA3 levels, as is shown in Fig. 3F (r = 0.63, p < 0.001).

### uSerpinA3 in CKD secondary to lupus nephritis

**Figure 4:**
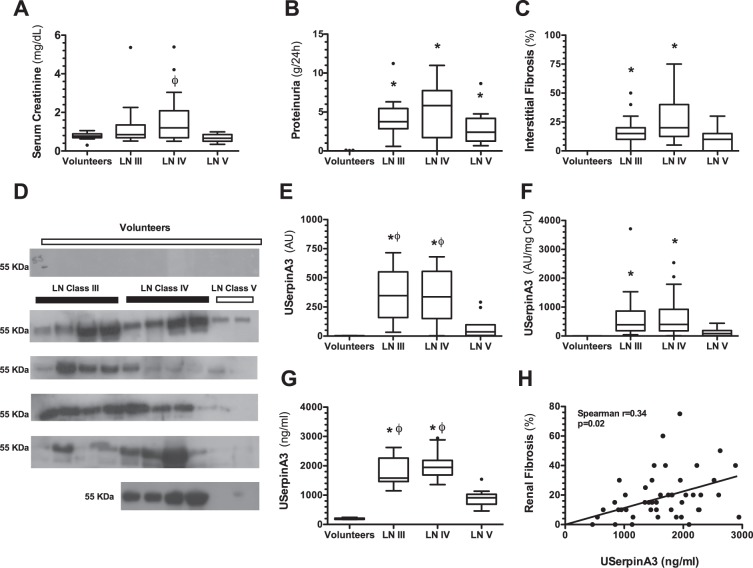
Renal clinical characteristics and urinary serpinA3 in patients diagnosed with LN. (**A**) Serum creatinine, (**B**) Proteinuria, (**C**) Percentage of renal fibrosis, and (**D**) WB autoradiography for urinary serpinA3 levels showing all the patients included. (**E**) Densitometric analysis for WB. (**F**) Urinary serpinA3 corrected by urinary creatinine. (**G**) Urinary serpinA3 levels determined by ELISA. (**H**) Spearman’s correlation of urinary serpinA3 and tubule-interstitial fibrosis. LN III = class III lupus nephritis (n = 18); LN IV = class IV lupus nephritis (n = 18); LN V = class V lupus nephritis (n = 11). Data are presented as Tukey’s box and whiskers plots. Healthy volunteers (n = 20), *p < 0.001 vs. Healthy volunteers, ^ϕ^p < 0.001 vs. class V LN.

To translate these findings to the clinical setting, we evaluated whether serpinA3 could be used as a biomarker in CKD patients. For this purpose, we analyzed serpinA3/Alpha-1-antichymotrypsin levels in patients diagnosed with class III, IV and V lupus nephritis, focal and segmental glomerulosclerosis (FSGS), ANCA associated vasculitis (AAV), and compared them with healthy volunteers. As these patients were diagnosed by kidney biopsy, CKD diagnosis was based on the histopathological analysis, severe proteinuria, and/or renal dysfunction. The patients with LN were classified using the ISN/RPS classification by a qualified nephropathologist. A representative microphotograph of hematoxylin/eosin staining and immunofluorescence that allowed the LN classification are shown in the Supp. Fig. 3. In this study, we included 47 LN patients of different classes and compared them with 20 healthy volunteers. The demographical characteristics of these patients are shown in Table 1. As expected in LN, the proportion of women was greater in all the groups and most were young patients. The time of evolution from systemic lupus erythematosus diagnosis and the time elapsed between the first renal symptom and the biopsy appears also in Table 1. In accordance with previous studies, the worst renal fibrosis and tubular atrophy was seen in class III and IV LN patients (proliferative LN)^5^. As shown in Fig. 4A, the serum creatinine levels were higher in class IV LN compared to class V patients (membranous LN). All LN patients exhibited severe proteinuria and variable renal fibrosis degrees (Fig. 4B,C). uSerpinA3 was not detected in the urine from healthy volunteers, but it was significantly elevated in classes III/IV LN patients, whereas class V LN group exhibited lower uSerpinA3 levels as is shown by the Western blot analysis in Fig. 4D. The absolute uSerpinA3 values are presented in Fig. 4E and the uSerpinA3 corrected by urinary creatinine in Fig. 4F. The results obtained by ELISA were similar to those by Western blot analysis (Fig. 4G). There were no differences in plasma serpinA3/Alpha-1-antichymotrypsin levels among LN groups and between LN and healthy volunteers (Supp. Fig. 4A) and there was no correlation between plasma and uSerpinA3 levels (Supp. Fig. 4B). As found in the rat model, we observed a significant correlation between the uSerpinA3 levels (ELISA) and interstitial fibrosis % (r = 0.34, p = 0.02, Fig. 4H). Moreover, uSerpinA3 levels discriminated between class III/ IV LN patients from class V LN, with a c-statistic of 0.85 (Supp. Fig. 5). We constructed a linear regression model to determine factors associated to renal interstitial fibrosis (dependent variable). The model included age, serum creatinine, proteinuria, histological activity score, plasma and urinary SerpinA3 (Suppl. Table 2). Urinary SerpinA3, serum creatinine and proteinuria were independently associated to the degree of interstitial fibrosis. There was no collinearity among the included variables (VIF < 1.4 for all variables).

**Table 1:** Clinical characteristics of the studied patients.

	Volunteers n = 20	Class III LN n = 18	Class IV LN n = 18	Class V LN n = 11	AAV n = 19	FSGS n = 14
Age, years	31 ± 10	31 ± 10	30 ± 11	33 ± 14	54 ± 15	36 ± 10
Female, n (%)	6 (30)	14 (75)	16 (89)	9 (82)	14 (74)	4 (29)
Months from SLE diagnosis, median (IQR)	—	23 (0–62)	36 (4–83)	27 (8–116)	—	—
Months from renal symptoms start, median (IQR)	—	2 (1–5)	2 (1–4)	3 (1–5)	1 (0–2)	6 (2–11)
Creatinine, mg/dl, median (IQR)	0.8 (0.7–0.9)	0.8 (0.7–1.3)	1.2 (0.7–2.1)^ϕ^	0.7 (0.5–0.9)	2.6 (1.2–3.6)*	0.9 (0.8–1.4)
eGFR, ml/min/1.73 m^2^, median (IQR)	118 (105–129)	99 (50–119)	64 (31–119)*	111 (103–132)	24 (14–90)*	85 (61–107)
Proteinuria, g/g, median (IQR)	0.1 (0.0–0.1)	2.7 (1.9–4.0)*	5.7 (3.4–8.4)*	2.9 (1.9–4.3)*	2.2 (0.7–3.1)*	3.3 (1.6–6.2)*
Hematuria, n (%)	0 (0)	12 (67)	17 (94)	7 (64)	18 (95)	11 (79)
+ve dsDNA antibodies, n (%)	ND	15 (83)	16 (89)	7 (64)	0 (0)	0 (0)
Complement C3, mg/dl	ND	73 (60–90)	53 (41–72)	111 (71–131)	119 (103–132)	148 (126–189)
Complement C4, mg/dl	ND	9 (8–14)	8 (8–14)	14 (11–18)	32 (20–42)	32 (30–46)
Interstitial fibrosis, %, median (IQR)	ND	15 (10–20)	20 (12.5–40)^ϕ^	10 (0–15)	30 (25–50)^+^	17.5 (10–40)
Tubular atrophy, %, median (IQR)	ND	15 (10–20)	20 (12.5–30)^ϕ^	10 (0–15)	30 (30–60)^+^	15 (15–40)

Abbreviations. LN, lupus nephritis; AAV, ANCA-associated vasculitis; FSGS, focal and segmental glomerulosclerosis; SLE, systemic lupus erythematosus; eGFR, estimated glomerular filtration rate calculated by the CKD-EPI formula; +ve dsDNA antibodies, positive antibodies directed to double-strand DNA; ND, not determined. Mean ± S.D.

^*^p < 0.05 vs. volunteers, ^Φ^p < 0.05 vs. class V LN, and ^+^p < 0.05 vs. FSGS.

**Figure 5:**
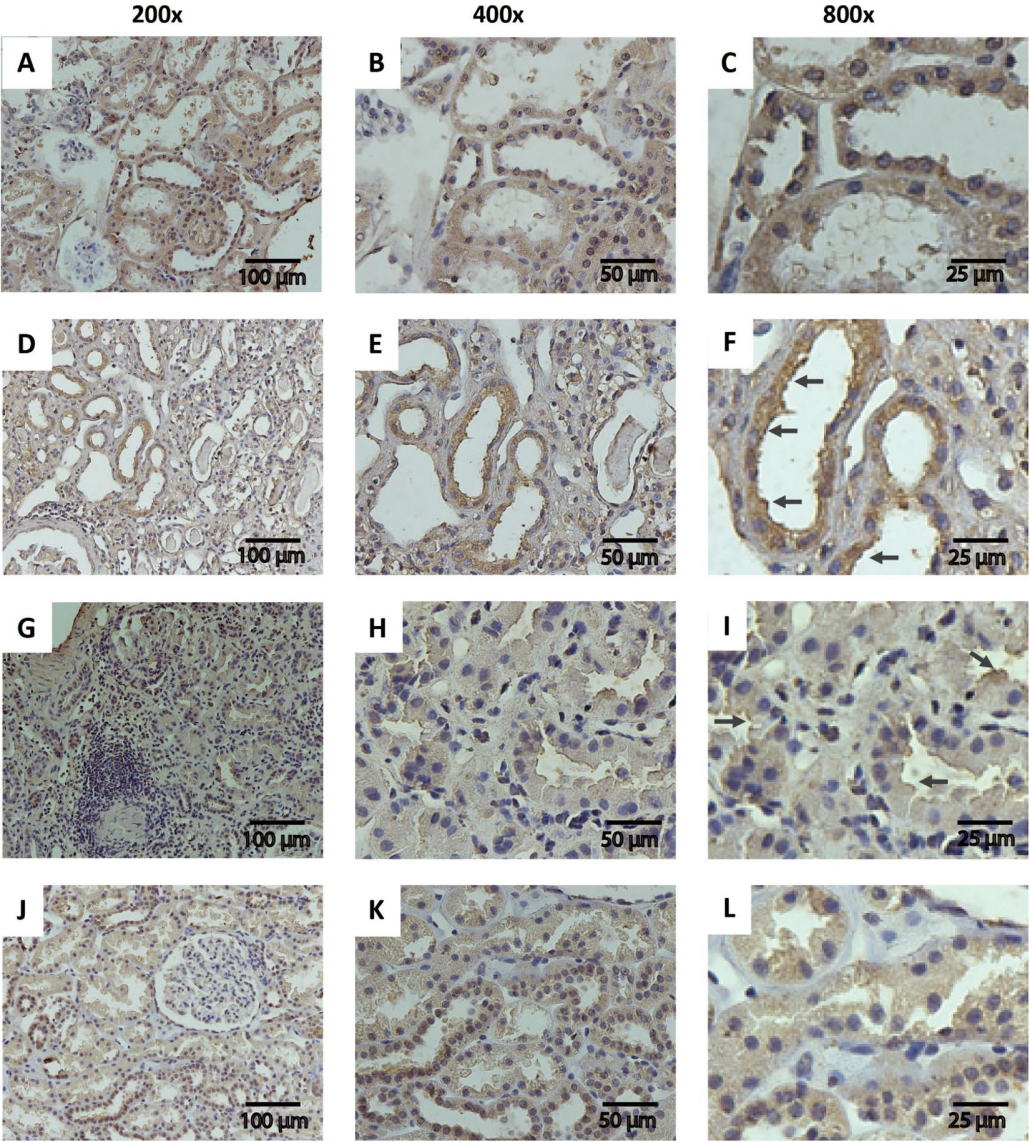
Serpin A3K immunohistochemistry in renal biopsies. (**A**–**C**) Representative micrographs of serpinA3 in a biopsy from healthy donor (Magnification 200x, 400x and 800x, respectively); (**D**–**F**) Representative micrographs of serpinA3 from a patient diagnosed with LN class III (Magnification 200x, 400x and 800x, respectively); (**G**–**I**) Representative micrographs of serpinA3 from a patient with LN class IV (Magnification 200x, 400x and 800x, respectively); (**J**–**L**) Representative micrographs of serpinA3 from patient with LN class V (Magnification 200x, 400x and 800x, respectively). Black arrows point the relocation to apical membrane in CKD patients.

In accordance to our experimental findings, serpinA3 was found in the cytosol of tubular epithelial cells from kidney donor’s biopsies (Fig. 5A–C). By contrast, in class III/IV LN patients, serpinA3 was relocated to the apical tubular membrane (Fig. 5D–I). Interestingly, class V LN patients, whom exhibited lower uSerpinA3 levels, the relocation was minimal (Fig. 5J–L).

### uSerpinA3 levels in CKD secondary to FSGS and AAV

To explore other etiologies of renal injury, we included patients diagnosed with FSGS and AAV. The Table 1 shows also the demographical characteristics of these two groups compared with the volunteers group. The AAV patients were older than the other groups and the percentage of women was higher in all the groups. The worst renal structural injury was seen in the AAV group.

**Figure 6:**
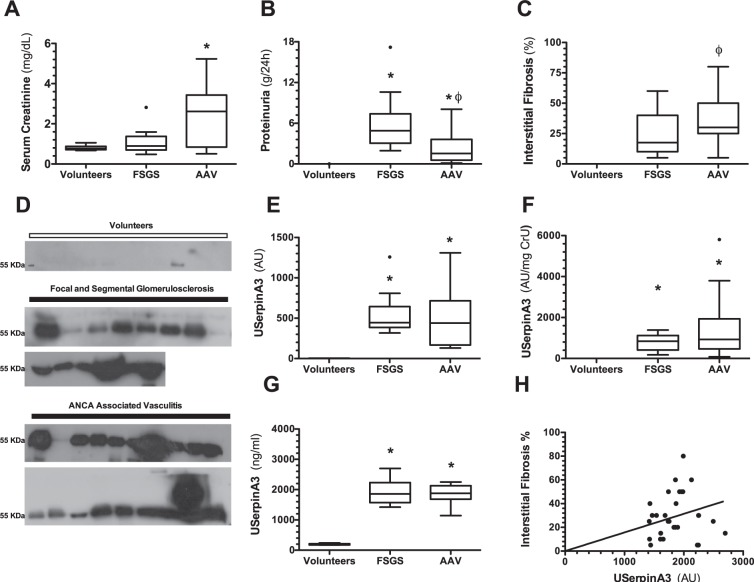
Renal clinical characteristics and urinary serpinA3 in patients diagnosed with FSGS and AAV. (**A**) Serum creatinine, (**B**) Proteinuria, (**C**) Percentage of renal fibrosis, and (**D**) WB autoradiography for urinary serpinA3 levels showing all the patients included. (**E**) Densitometric analysis for WB. (**F**) Urinary serpinA3 corrected by urinary creatinine. (**G**) Urinary serpinA3 levels determined by ELISA. (**H**) Spearman’s correlation of urinary serpinA3 and tubule-interstitial fibrosis. Focal Segmental Glomerulosclerosis = FSGS (n = 14); ANCA-associated vasculitis = AAV (n = 19). Data are presented as Tukey’s box and whiskers plots. *p < 0.001 vs. Healthy volunteers, ^ϕ^p < 0.001 vs. FSGS.

The AAV group exhibited severe renal injury characterized by a significant elevation of serum creatinine, proteinuria, renal fibrosis and tubular atrophy (Fig. 6A–C, Table 1). In the FSGS group, serum creatinine was not different from the volunteers group (Fig. 6A), but the proteinuria was more severe than in the AAV group (Fig. 6B), despite similar tubular interstitial fibrosis (Fig. 6C). As shown in Fig. 6D, uSerpinA3 was significantly higher in patients with FSGS and AAV compared to the volunteer’s group as is shown by the Western blot, the densitometric analysis (Fig. 6E) and the uSerpinA3 corrected by urine creatinine (Fig. 6F). All results obtained by Western blot were later confirmed by a commercial serpinA3/alpha-1-antichymotrypsin ELISA (Fig. 6G). As we could not determine renal fibrosis in healthy volunteers group, no significant correlation between uSerpinA3 levels and renal fibrosis % was found, but it can be appreciated that to greater fibrosis, uSerpinA3 levels were higher (Fig. 6H).

### uSerpinA3 levels in patients with inflammatory diseases without renal dysfunction

To determine the specificity of uSerpinA3 for detecting kidney injury, we evaluated uSerpinA3/alpha-1-antichemotrypsin in patients with inflammatory diseases, but without renal dysfunction. For this purpose, patients with liver cirrhosis (LC), acute pancreatitis (AP) and active rheumatoid arthritis (RA) were included. The clinical characteristics of these patients are shown in the Suppl. Table 1. As shown in the Suppl. Fig. 6A,B, uSerpinA3 was not detected in LC, AP, and RA groups by either Western blot or ELISA.

## Discusion

Using high-resolution mass spectrometry, we isolated urine proteins from animals with CKD. Among the identified proteins were: albumin, serpinA3, serotransferrin, alpha-1-antiproteinase, serine A3M protease inhibitor, LOC299282 protein, serpinC1, kininogen T, B-fetuin, and type I keratin. Albumin and serpinA3 constituted the most abundant proteins.

Serpins are a family of serine protease inhibitors. Thirty-four serpins from nine classes have been identified and characterized in humans to date; within these proteins is the serpinA3/Alpha-1-Antichymotrypsin (serine-proteinase inhibitor, class A, member 3), whereas the homologous for rodents is serpinA3K^23^. The genecards website, a database of human genes (http://www.genecards.org/cgi-bin/carddisp.pl?gene=SERPINA3&keywords=serpinA3), has reported serpinA3 expression in different tissues, evaluated by three different approaches: RNAseq, microarrays and a serial analysis of gene expression (SAGE), with the largest expression in retina, kidney, liver, and pancreas (Suppl. Fig. 7). In the kidney, Fleming S. *et*.*al*.^24^ identified serpinA3 in the tubular proximal epithelium, the mesenchyme of nephroblastomas and the adult renal cell carcinoma. Similarly, Khan T.N. *et al*.^25^ found serpinA3 immunostaining in proximal tubules from control biopsies. We confirmed these findings in this work showing that in the kidney from control rats and in kidney from donor biopsies, serpinA3 is mainly located in the cytoplasm of tubular epithelial cells (Figs 3C,D and 5A–C).

SerpinA3 has been involved in different pathologies such as hypertension, inflammation, and angiogenesis^26^. Specifically, in cornea and retina, serpinA3 promotes anti-inflammatory, anti-angiogenic, anti-oxidant and anti-fibrotic actions^26–30^. However, little is known about the specific role of serpinA3 in the renal pathophysiology. One study showed increased serpinA3 staining in the proximal renal tubules in biopsies from a variety of primary and secondary glomerulonephritis, such as minimal-change disease (MCD), FSGS, diffuse mesangial proliferative glomerulonephritis (MeGN), membranous glomerulonephritis (MGN), diabetic nephropathy, IgA nephritis and LN compared to normal renal tissues^31^.

In the present study, using a model of AKI to CKD transition in rats, we found that serpinA3 appears in the urine since the first month post-ischemia, when the rats do not even exhibit proteinuria or any sign of glomerular damage. Moreover, uSerpinA3 levels positively correlated with renal fibrosis, hence higher uSerpinA3 levels associate with higher renal tissue fibrosis.

To address if urinary serpinA3 was being filtrated, we measured plasma serpinA3 and found no correlation between plasma serpinA3 with urinary serpinA3 levels. Furthermore, plasma serpinA3 levels were similar in healthy volunteers and among the different lupus nephritis classes (Suppl. Fig. 5) and uSerpinA3 levels were independently associated with interstitial fibrosis. We cannot fully exclude that some fraction of serpinA3 may be filtered in pathophysiological conditions, however, in our rat model, we showed that serpinA3 appears in the urine since the first month post renal ischemia, much earlier than proteinuria. Furthermore, the subgroup of class V LN patients with higher degrees of proteinuria (>3 g per day) still exhibited minimal serpinA3 urine excretion. As serpinA3 is mainly expressed in organs such as liver or pancreas, we also included patients with acute inflammation (pancreatitis) or chronic damage (cirrhosis). Urinary serpinA3 levels were not modified in these conditions. These evidences together strongly suggest that most of the serpinA3 found in the urine has renal origin.

In pathologic conditions, serpinA3 was relocated from the cytoplasm to the apical epithelial membrane (Fig. 3). This suggests: 1) that uSerpinA3 reflects intra-renal injury, 2) that during renal injury, serpinA3 is probably secreted into the luminal space explaining its emergence in the urine, and 3) that uSerpinA3 is an early and timely marker of AKI to CKD transition.

We then analyzed the expression of uSerpinA3 in human renal diseases. We showed that uSerpinA3 is not detectable in healthy volunteers but it increases in kidney diseases from different etiologies: LN, FSGS and AAV. In LN patients, uSerpinA3 levels were found elevated in severe classes III/IV LN that are usually associated with greater kidney fibrosis, and less elevated, in the less inflammatory class V LN that usually carries better prognosis^32^. It has also been reported increased uSerpinA3 levels in active LN patients but not in systemically active lupus without renal involvement^33^. These suggests that uSerpinA3 derives from kidney and its elevation is probably associated with higher inflammation and fibrosis. However, the potential use of uSerpinA3 as a biomarker needs further evaluation in larger studies.

SerpinA3 has been described to have anti-inflammatory properties^8–12,26,28^, therefore, we decided to evaluate uSerpinA3 in AAV, a severe renal inflammatory disease, and compared it to FSGS patients, a proteinuric but less inflammatory disease. Urinary SerpinA3 levels were equally elevated in both groups despite different degrees of proteinuria and positively correlated with the percentage of renal fibrosis in the biopsy. Furthermore, we studied patients with extrarenal inflammatory diseases (rheumatoid arthritis, pancreatitis, liver cirrhosis) with preserved renal function. Urinary SerpinA3 levels were similar in these patients compared to those observed in healthy volunteers. This also supports that uSerpinA3 is kidney-derived and probably more related to fibrosis than to sytemuc inflammation.

The specific role of serpins in the renal physiology or pathophysiology has not been studied. Zhang B. *et al*.^27,29,30^, Liu X. *et al*.^26^ and Hu, J. *et al*.^28^ have demonstrated that serpinA3 blocks reactive oxygen species (ROS) generation, inflammation and pro-fibrotic pathways in the cornea and retina. Thus, our results suggest that serpinA3’s role in the kidney could be to counterbalance the inflammation, oxidative stress and renal fibrosis that follows renal damage. This hypothesis will require further study. As it is possible that serpinA3 may have a different role depending on the pathophysiological scenery, we need to wait for serpinA3’s transgenic mice that will be soon available to more profoundly evaluate its role in AKI and CKD.

The limitations of the present study are: 1) uSerpinA3 levels were measured in biological samples at a single time point, and further work has to be undertaken to evaluate the course of this protein longitudinally, and 2) although there was a good correlation with kidney fibrosis, it cannot be ignored that uSerpinA3 levels could be increased secondary to other ongoing inflammatory processes in the kidney.

This study supports that uSerpinA3 is an early and effective biomarker for the detection of renal injury, with a great potential to be used in the diagnosis of the AKI to CKD transition and CKD from different etiologies. Our observations in humans suggest that uSerpinA3 can be a useful marker for renal fibrosis and inflammation, with a potential role to differentiate between class III/IV and class V lupus nephritis.

## Supplementary information

Supplemental Information

Supplemental Western Blots
