# Supplementary material for: SerpinA3 in the Early Recognition of Acute Kidney Injury to Chronic Kidney Disease (CKD) transition in the rat and its Potentiality in the Recognition of Patients with CKD
Source: Sci Rep. 2019 Jul 17;9:10350. doi: 10.1038/s41598-019-46601-1 (PMC6637202; doi:10.1038/s41598-019-46601-1)
Supplement: Supplementary file 1 — Supplemental Information [file 41598_2019_46601_MOESM1_ESM.pdf]

SerpinA3 in the Early Recognition of Acute Kidney Injury to Chronic Kidney Disease (CKD) transition in the rat and its Potentiality in the Recognition of Patients with CKD.

Andrea Sánchez-Navarro[1,2], Juan M. Mejía-Vilet[2], Rosalba Pérez-Villalva[1,2], Diego L. Carrillo-Pérez[4,5], Brenda Marquina-Castillo[3], Gerardo Gamba[,2,5], and Norma A. Bobadilla [1,2]

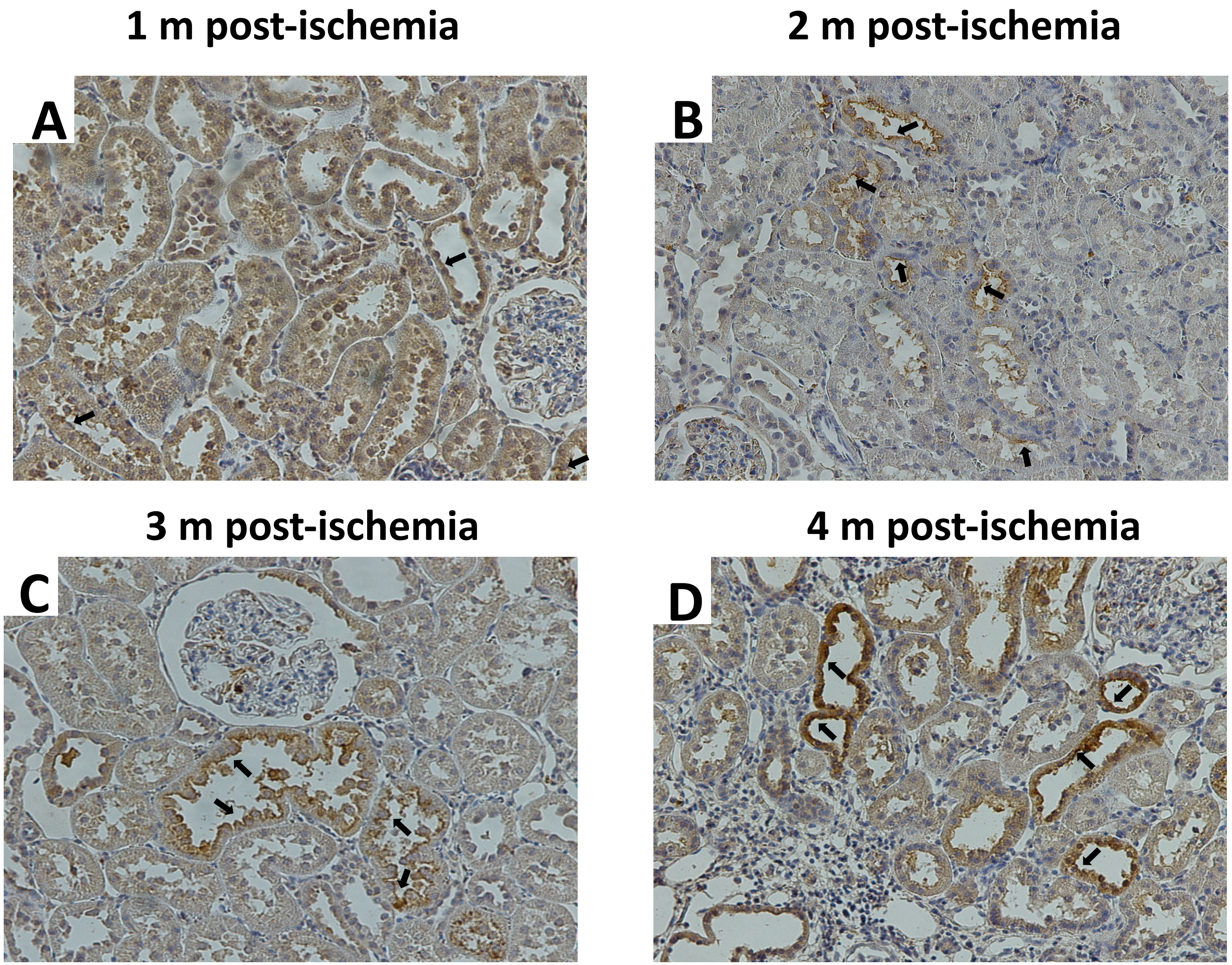

Supplemental Figure 1. SerpinA3 immunolocalization along the AKI to CKD transition. Representative microphotographs of serpinA3 immunostaining in renal cortex from rats euthanized A) 1, B) 2, C) 3 or D) 4 months after renal ischemia, magnification 800x. Black arrows show serpinA3 apical membrane immunostaining.

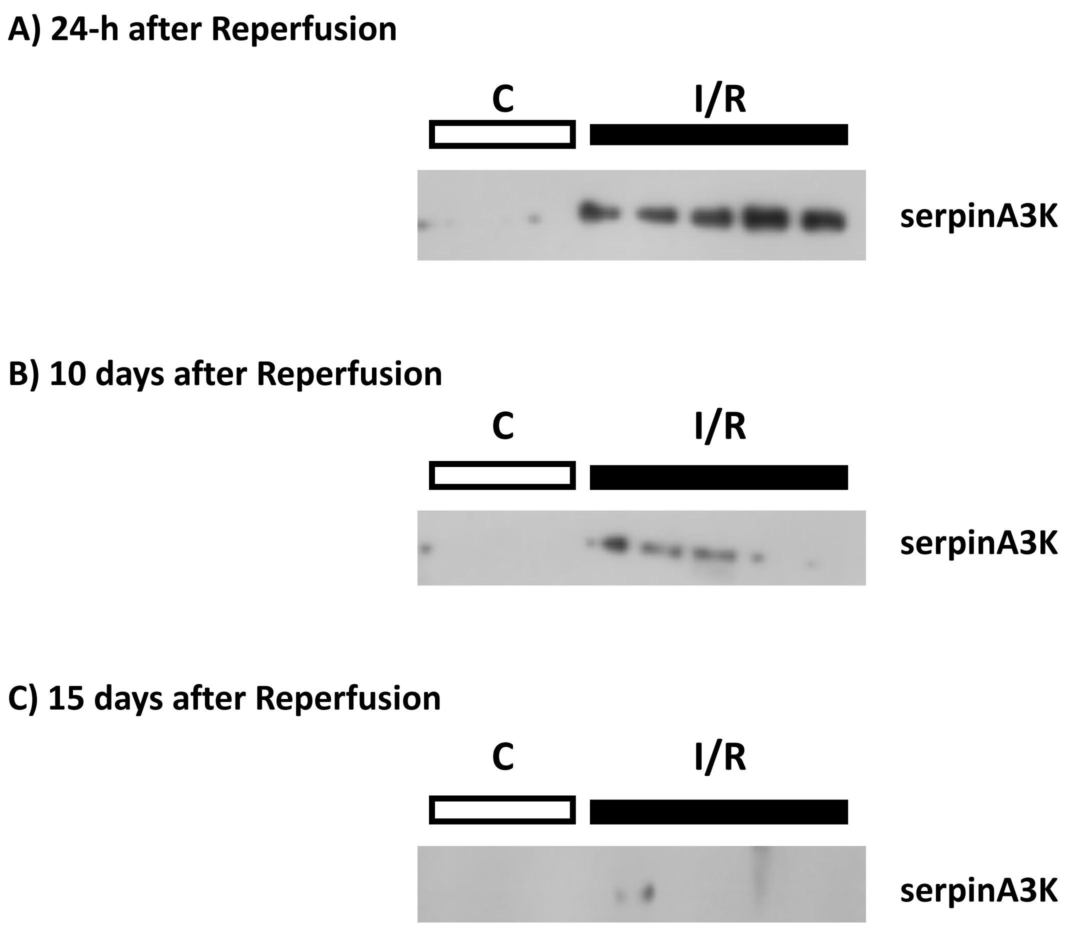

Supplemental Figure 2. After AKI resolution, uSerpinA3K is not detected in urine samples. Western Blot analysis showing serpinA3K in urine from control and I/R groups: A) after 24-h of reperfusion, B) 10 days after reperfusion, or C) 15 days after reperfusion.

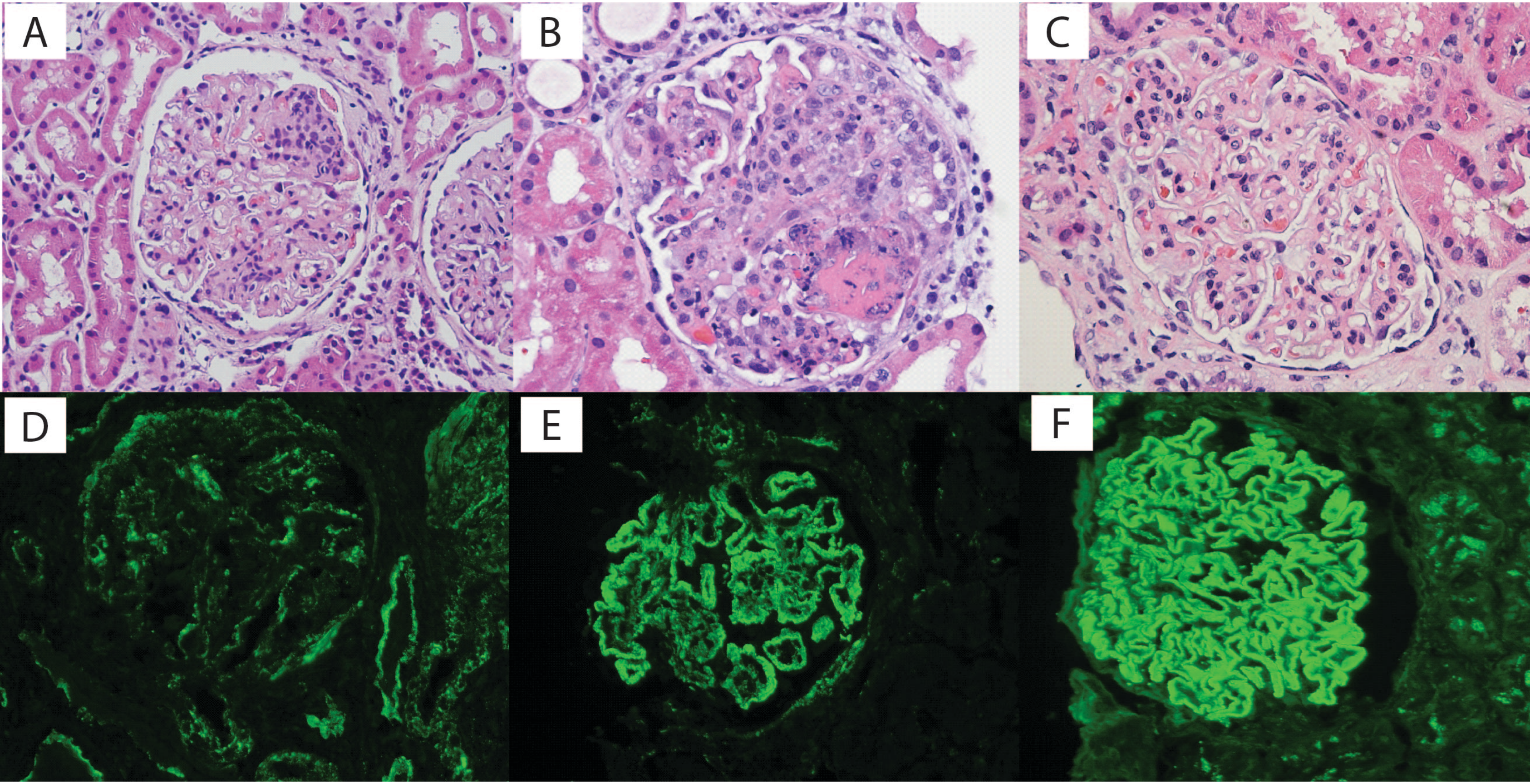

Supplemental Figure 3. ISN/RPS lupus nephritis classification representative images. A) Representative microphotograph of a class III LN patient characterized by immune complex deposition and leukocyte infiltration in <50% of glomeruli (HE staining), B) Class IV LN microphotograph showing immune complex deposition and leukocyte infiltration in >50% of glomeruli, C) Class V LN microphotograph exhibiting immune complex deposition exclusively in the subepithelial space without leukocyte infiltration. D) A representative immunofluorescence image showing C1q deposition in the mesangial and subepithelial spaces, E) shows C1q deposition in the mesangial and subepithelial spaces, and F) shows IgG immunofluorescence in the subepithelial space.

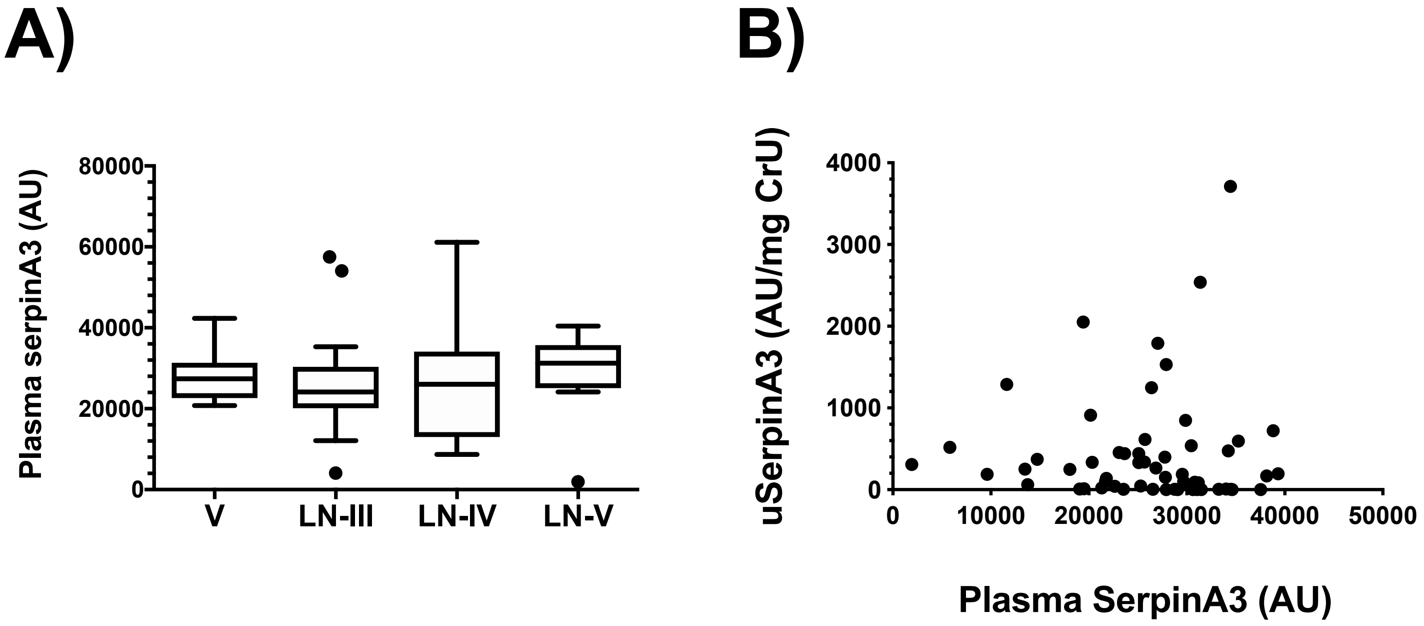

Supplemental Figure 4. Plasma serpinA3 levels and its correlation with uSerpinA3. A) Plasma SerpinA3 levels were not different among volunteers and lupus nephritis groups**.** B) There was no correlation between plasma and uSerpinA3 levels. Healthy volunteer = V; class III lupus nephritis = LN-III; class IV lupus nephritis = LN-IV; class V lupus nephritis = LN-V

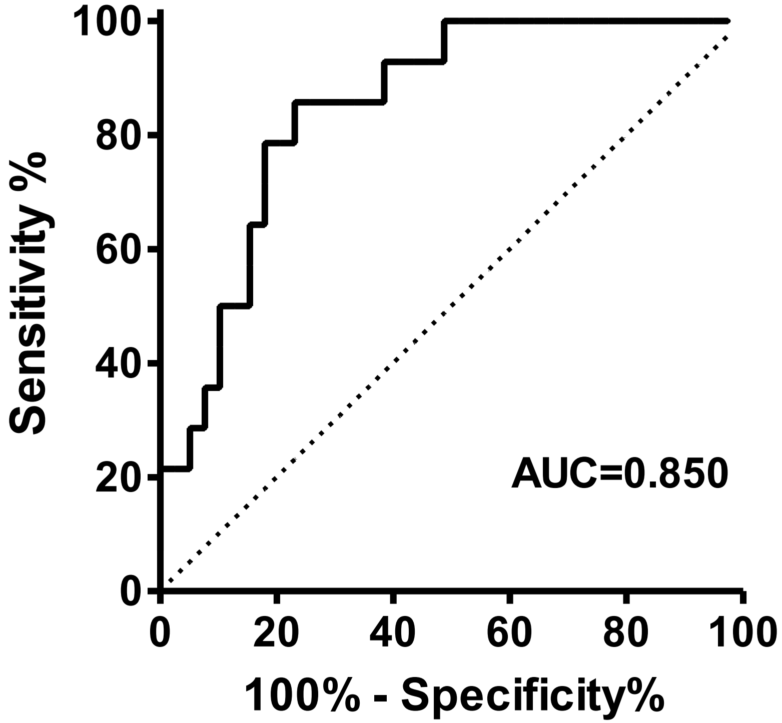

Supplemental Figure 5. Sensitivity and specificity of urine serpinA3K in different classes of LN. ROC analysis showing the ability of urine serpinA3K to discriminate class III/IV LN (proliferative) from class V LN (membranous). For a uSerpinA3K levels cutoff of 189.8 OD units/mg UCr, sensitivity was 85.7% and specificity 76.9% to discriminate between these LN classes.

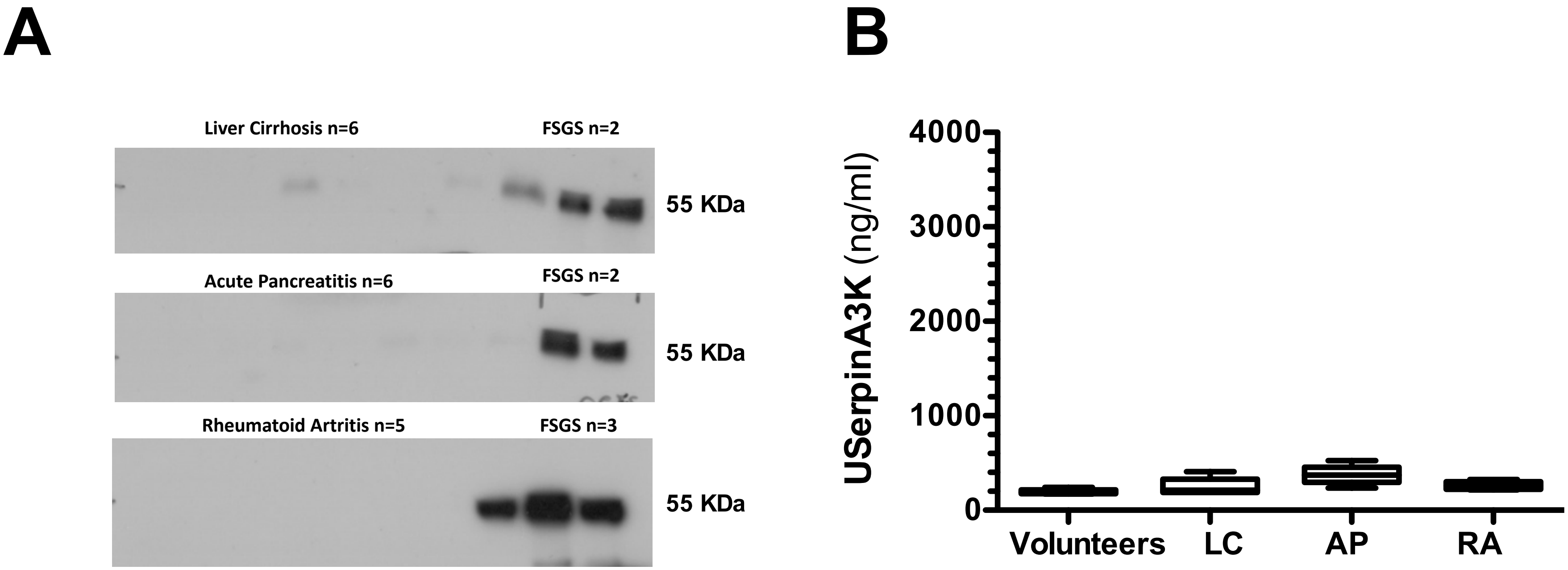

Supplemental Figure 6. The uSerpinA3K is not detected in patients with inflammatory diseases without kidney damage. A) Western blot of uSerpinA3K of all the patients. B) uSerpinA3K levels by ELISA. Liver Cirrhosis = LC; Acute Pancreatitis = AP; Rheumatoid Arthritis = RA.

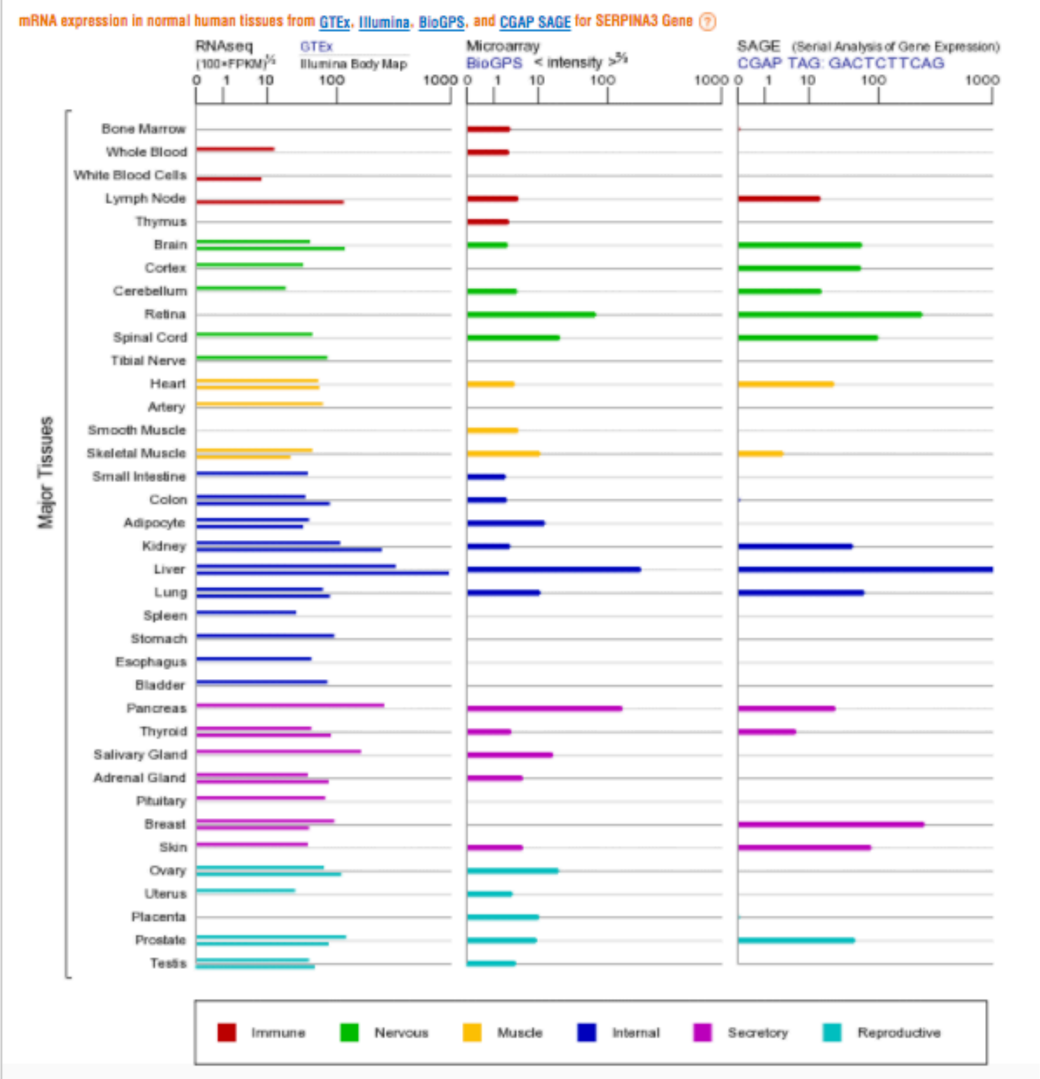

Supplemental Figure 7. Comparative mRNA serpinA3K expression in different tissues reported in the genecards website, (http://www.genecards.org/cgi-bin/carddisp.pl?gene=SERPINA3&keywords=serpinA3). The serpinA3K expression was evaluated by three different approaches: RNA seq, microarrays and a serial analysis of gene expression (SAGE).

Supplemental Table 1. Clinical Characteristics of Patients with normal renal function.

	Liver Cirrhosis (n=6)	Acute Pancreatitis (n=6)	Rheumatoid Arthritis (n=5)
Age (years)	56 (49-63)	41 (18-65)	41 (21-58)
Female Gender (%)	4 (66.6)	1 (16.6)	4 (80)
SCr (mg/dL)	0.7 (0.6-0.9)	0.7 (0.5-0.9)	0.6 (0.4-0.7)

Supplemental Table 2. Linear regression model exploring factors associated to interstitial fibrosis

Factor	B	p-value	VIF
Constant	-4.215	0.471	-
Age	0.006	0.743	1.086
log_2 creatinine	0.644	0.009	1.186
log_2 proteinuria	0.697	0.003	1.376
log_2 plasma SerpinA3	0.360	0.361	1.193
log_2 urinary SerpinA3	0.377	0.044	1.071
log_2 activity score	-0.036	0.426	1.385
