# Supplementary material for: SerpinA3 in the Early Recognition of Acute Kidney Injury to Chronic Kidney Disease (CKD) transition in the rat and its Potentiality in the Recognition of Patients with CKD
Source: Sci Rep. 2019 Jul 17;9:10350. doi: 10.1038/s41598-019-46601-1 (PMC6637202; doi:10.1038/s41598-019-46601-1)

SerpinA3 in the Early Recognition of Acute Kidney Injury to Chronic Kidney Disease (CKD) transition in the rat and its Potentiality in the Recognition of Patients with CKD.

Andrea Sánchez-Navarro1,2, Juan M. Mejía-Vilet2, Rosalba Pérez-Villalva1,2, Diego L. Carrillo-Pérez4,5, Brenda Marquina-Castillo3, Gerardo Gamba,2,5, and Norma A. Bobadilla 1,2

1Molecular Physiology Unit, Instituto de Investigaciones Biomédicas, Universidad Nacional Autónoma de México, 2Department of Nephrology and Mineral Metabolism, 3Department of Experimental Pathology and 4 Deparment of Internal Medicine, Instituto Nacional de Ciencias Médicas y Nutrición Salvador Zubirán, and 5Tecnológico de Monterrey, Escuela de Medicina y Ciencias de la Salud, Mexico City.

Western Blots

Figure 3B

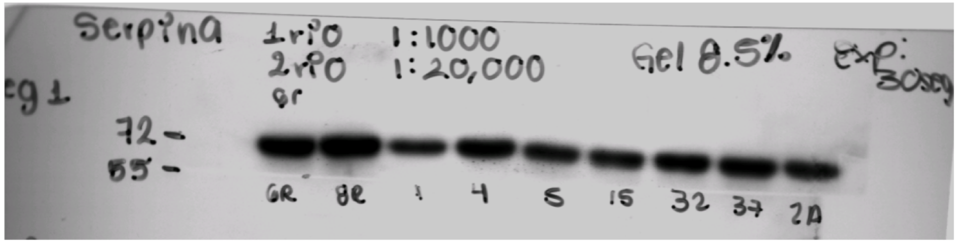

30 sec of exposure

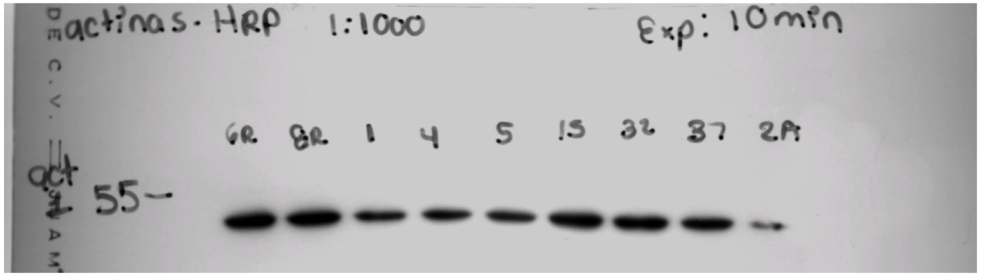

Figure 3C

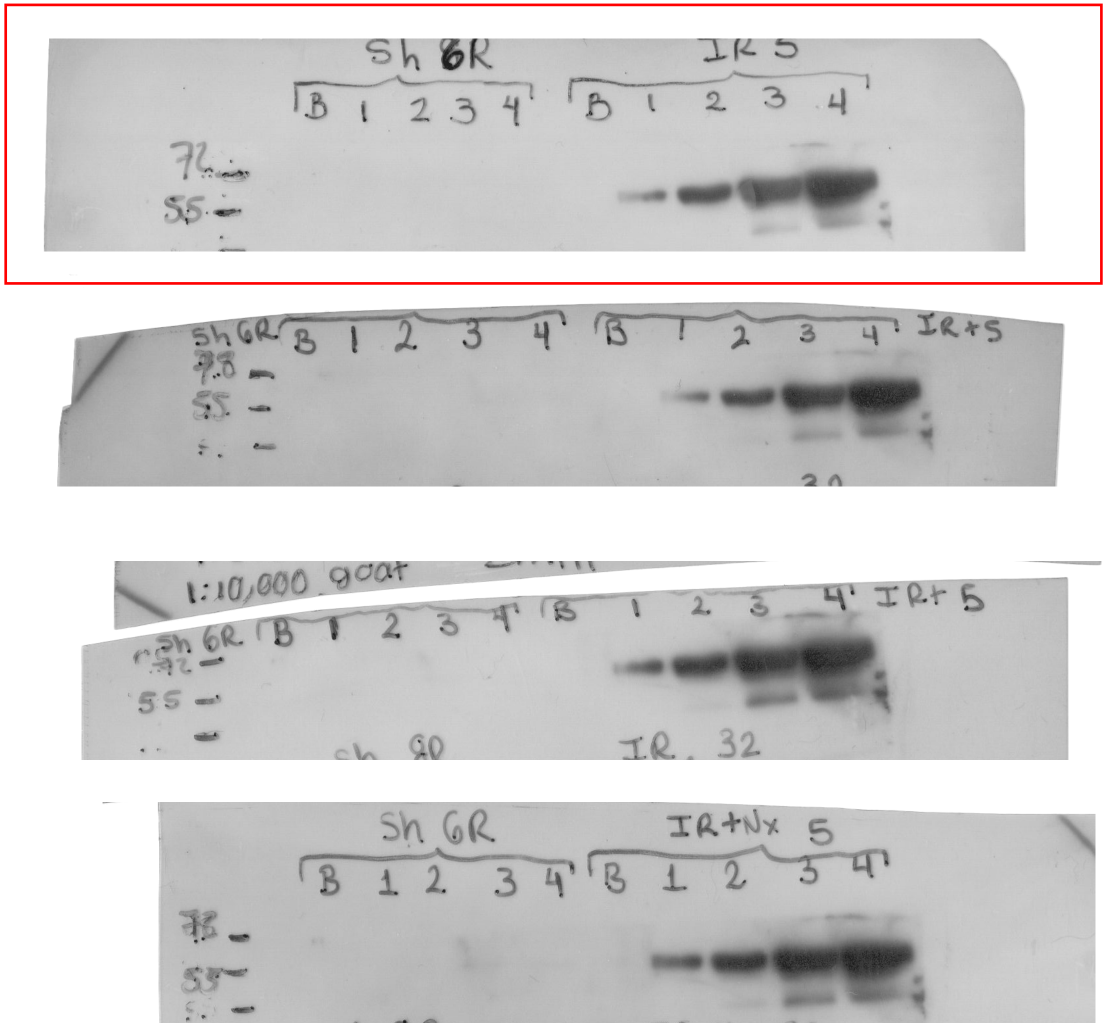

1min

2min

3min

5min

Figure 3C Blots of the rest of animals included

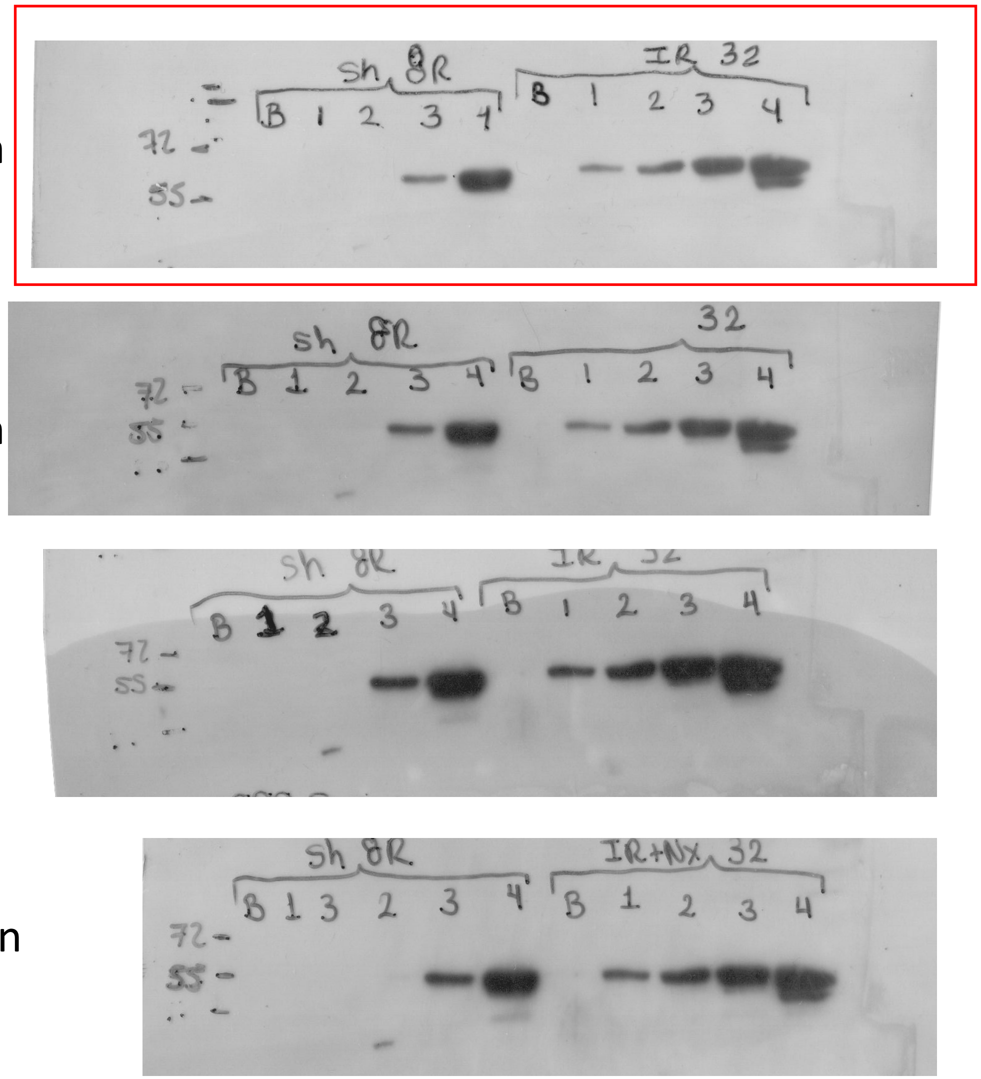

1min

2min

3min

5min

Figure 3C Blots of the rest of animals included

1min

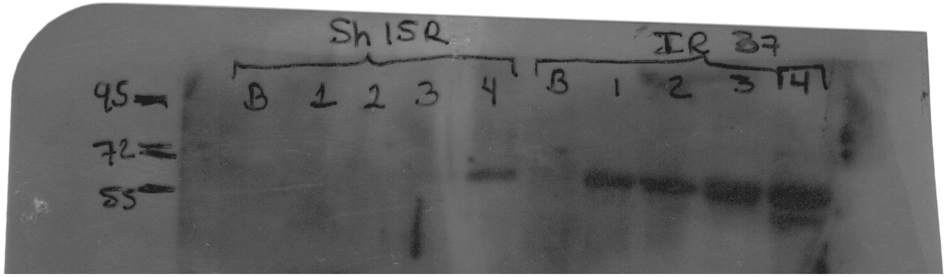

2min

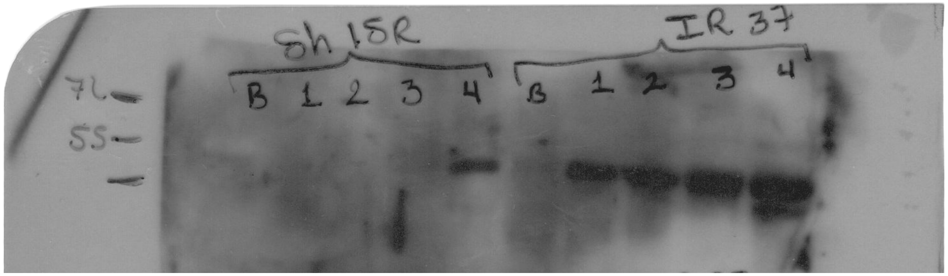

Figure 3C Blots of the rest of animals included

1min

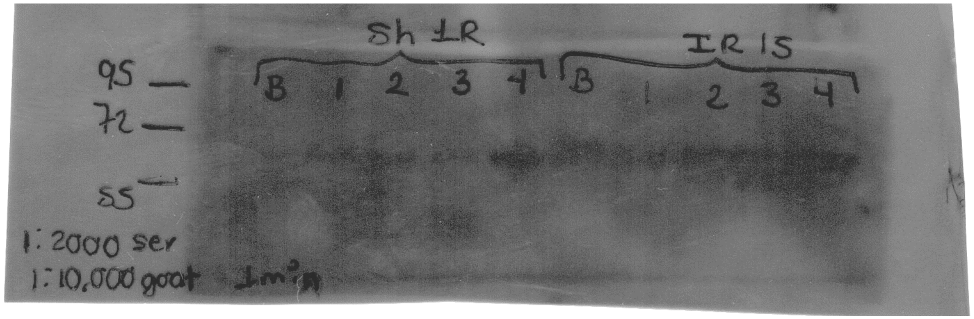

2min

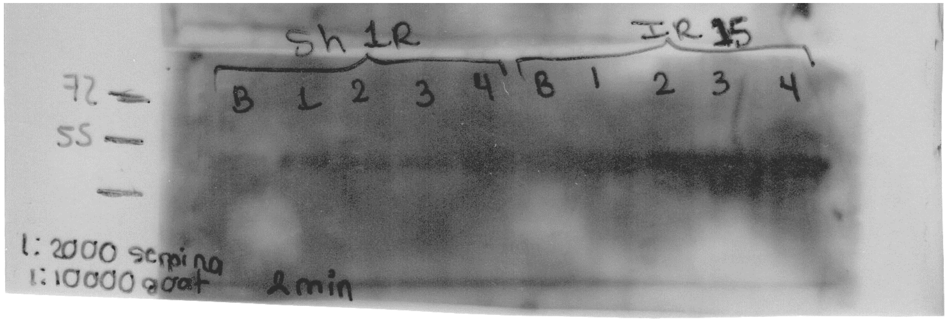

Figure 4D

Gel 1

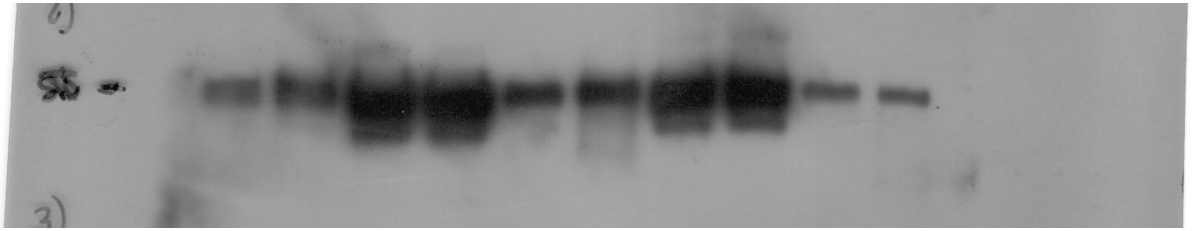

30sec

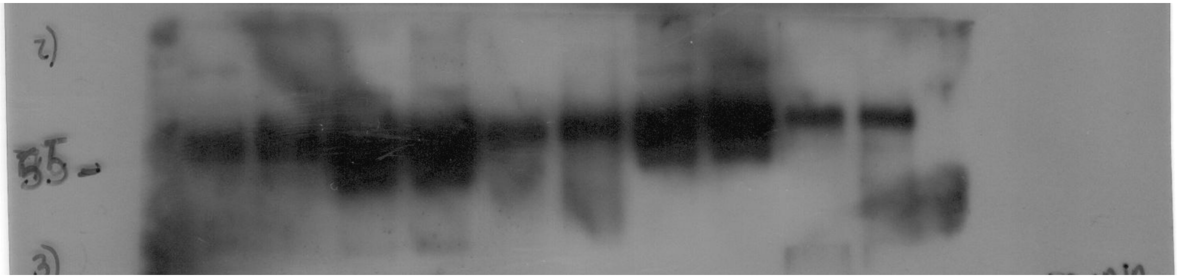

1min

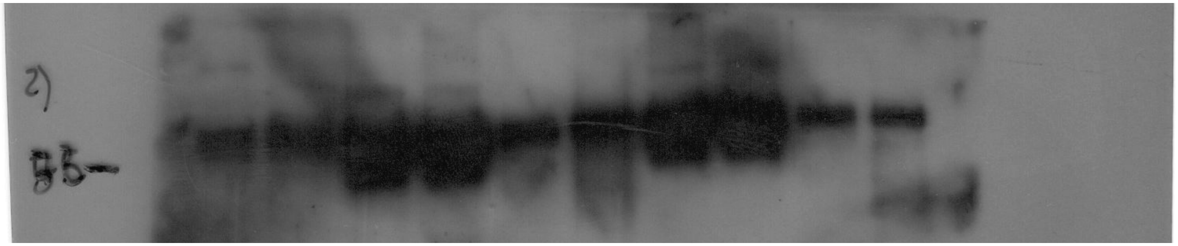

3min

Figure 4D

Gel 2

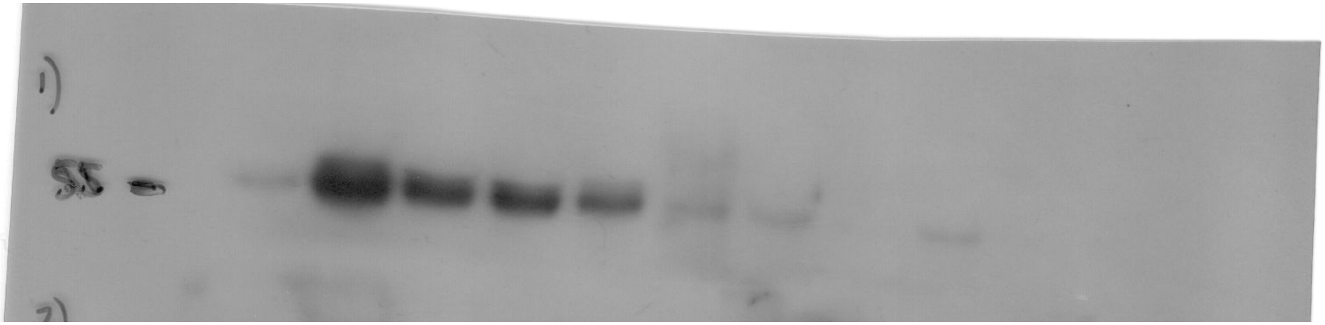

30sec

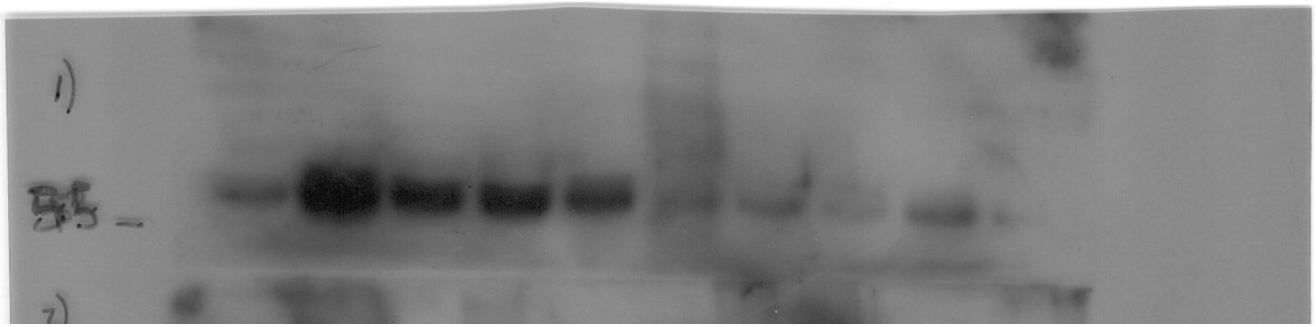

1min

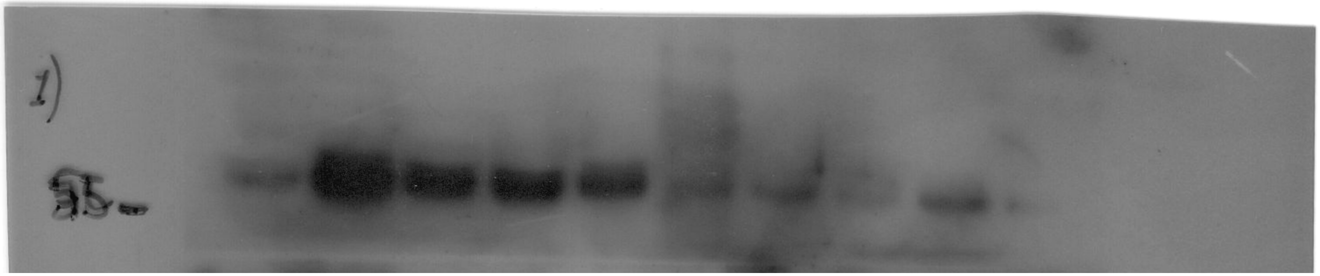

3min

The exposure of 1 minute was use for this figure.

Figure 4D

Gel 3

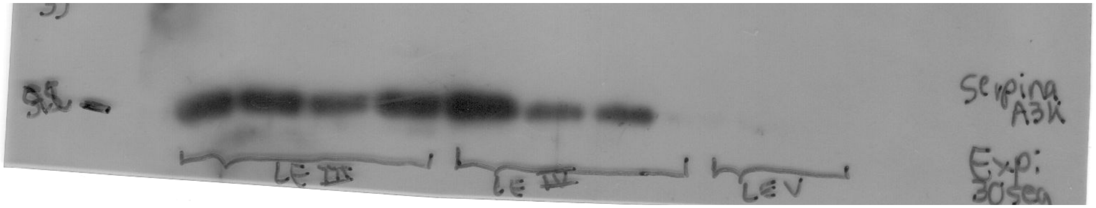

30sec

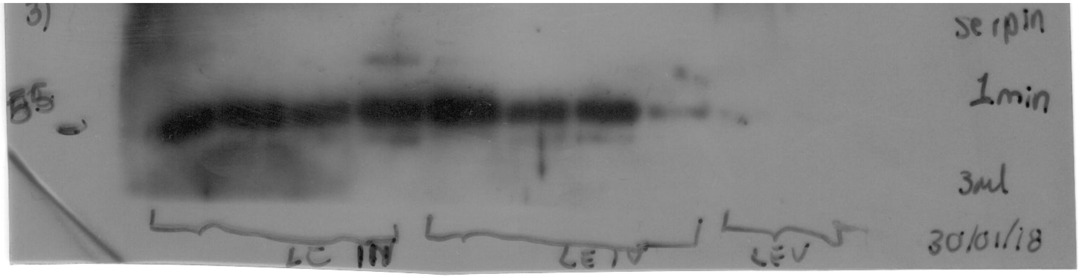

1min

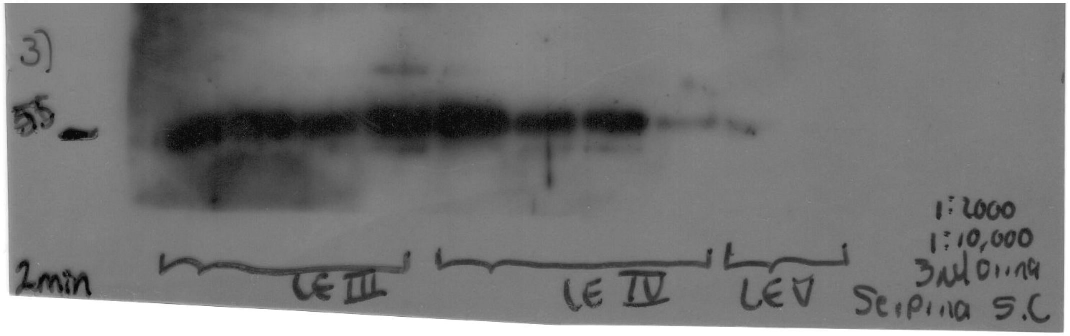

3min

Figure 4D

Gel 4

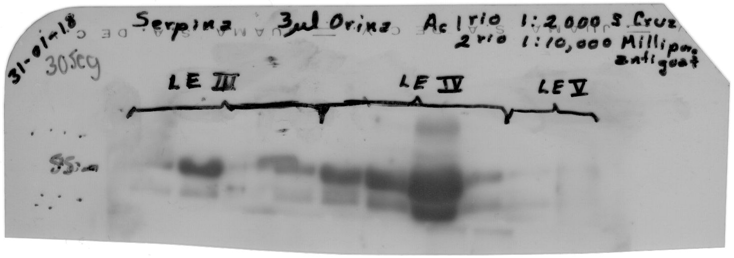

1min

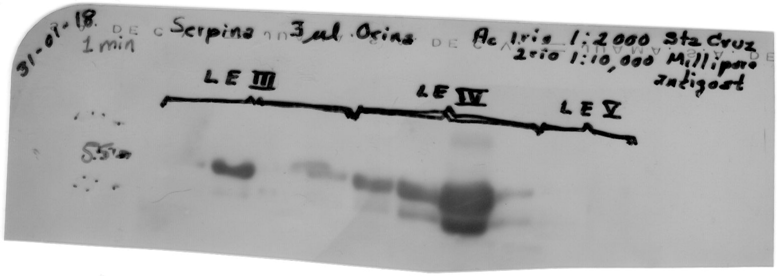

30sec

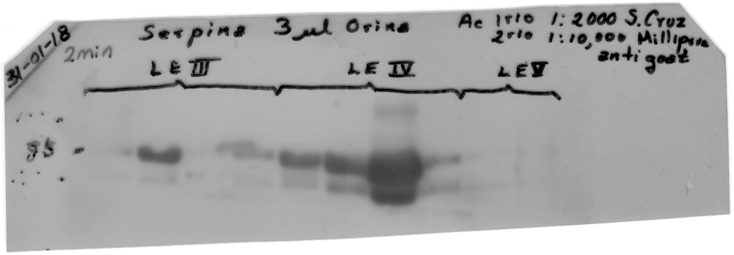

2min

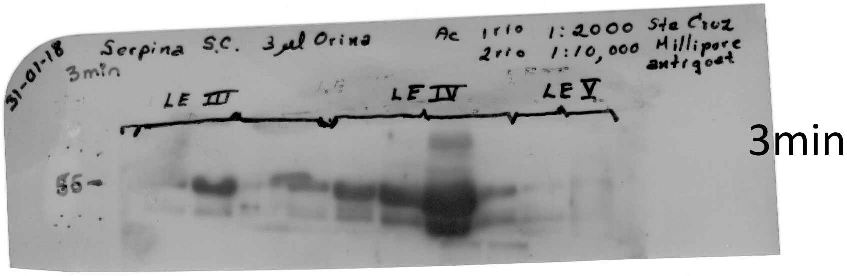

3min

Figura 4D

Gel 5

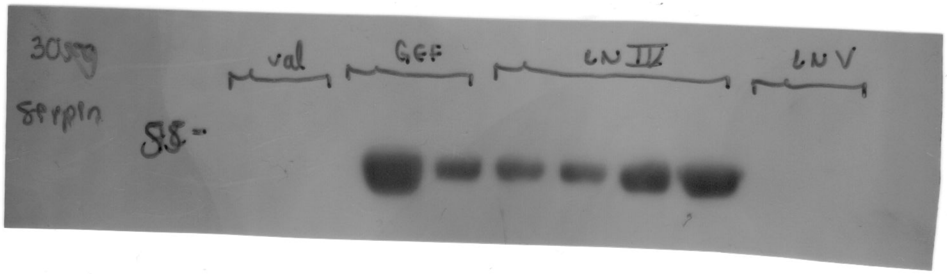

30sec

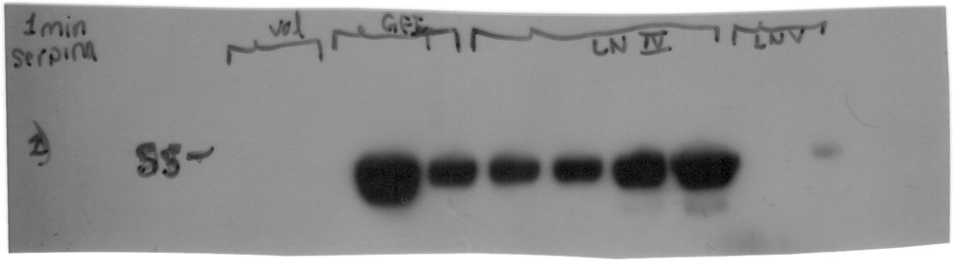

1min

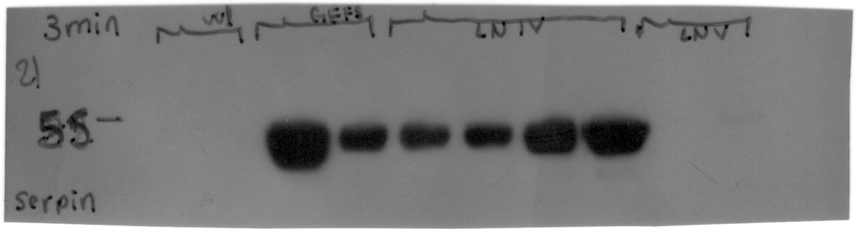

3min

Figure 6D

Gel 2

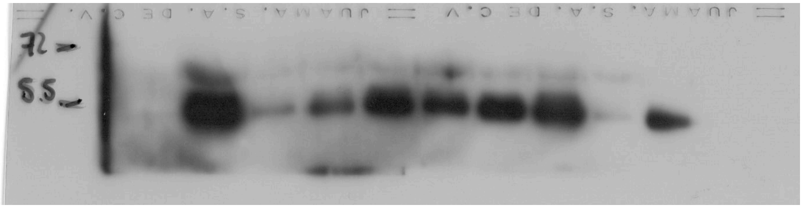

1min

Gel 3

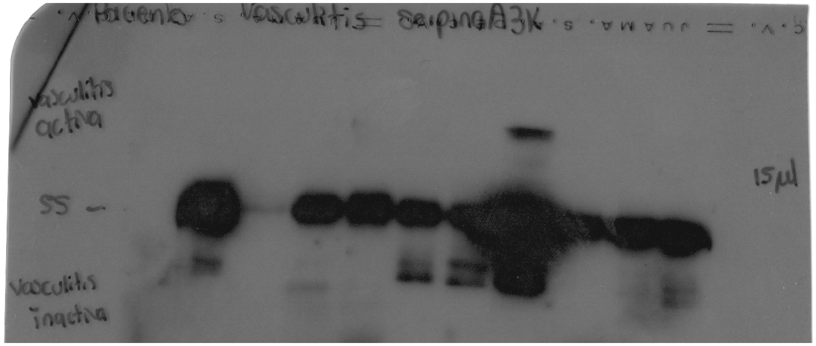

1min

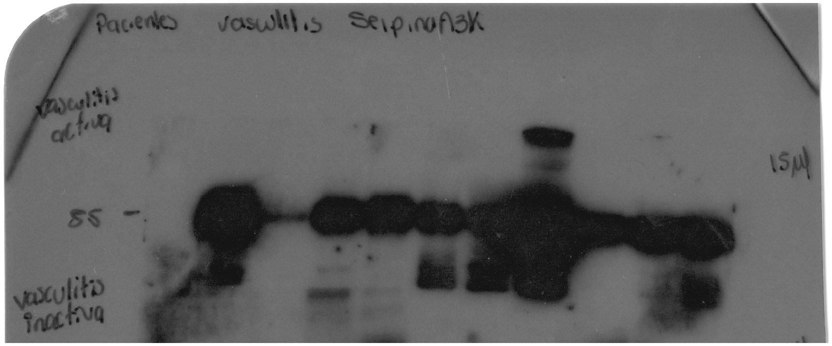

3min

Gel 4

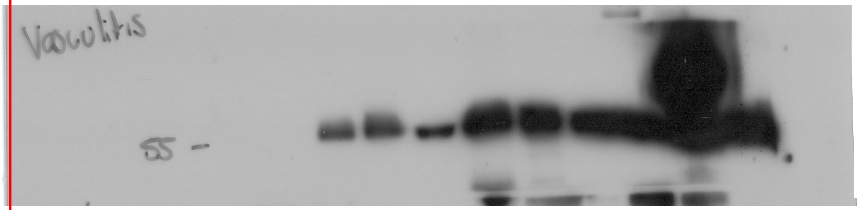

1min

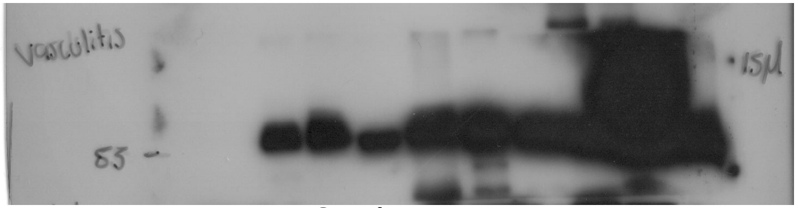

3min

Supplemental Figure 1A

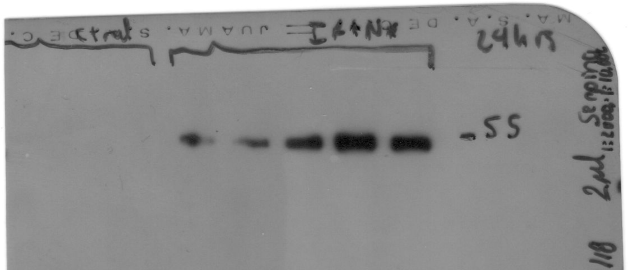

1min

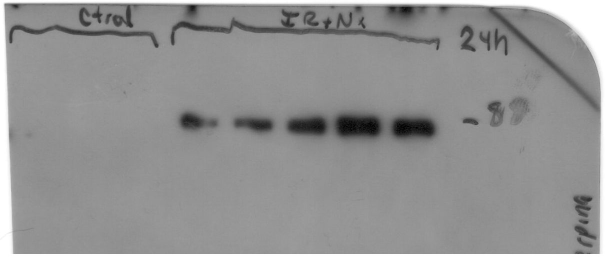

2min

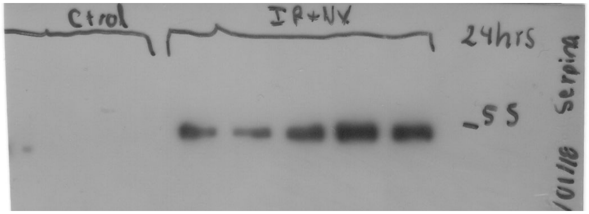

3min

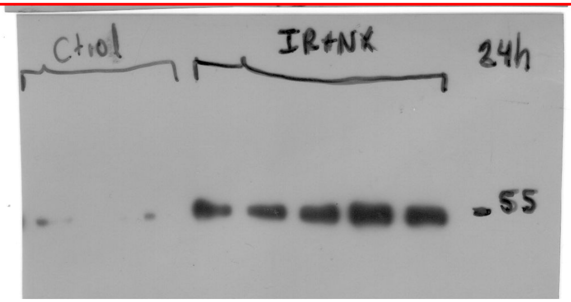

5min

The exposure of 5 minute was use for this figure.

Supplemental Figure 1B

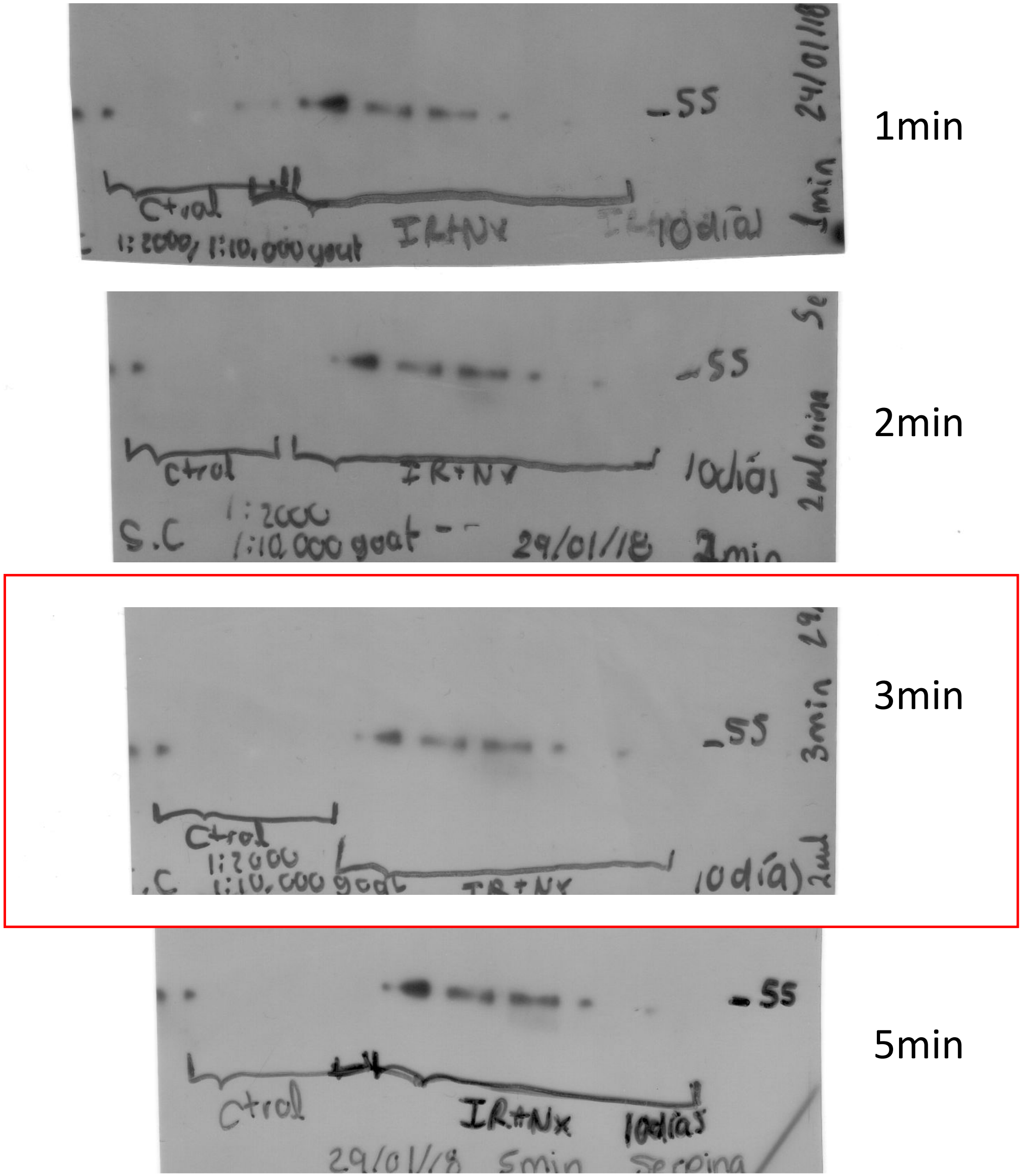

The exposure of 3 minute was use for this figure.

Supplemental Figure 1C

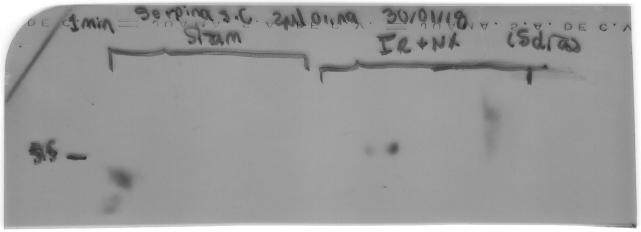

1min

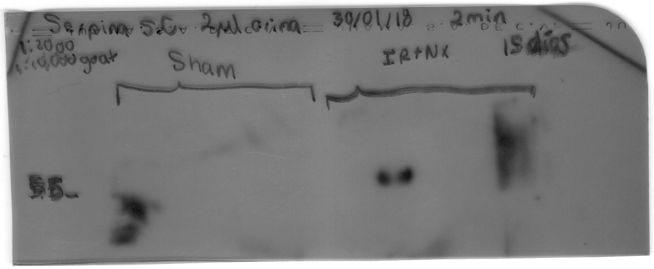

2min

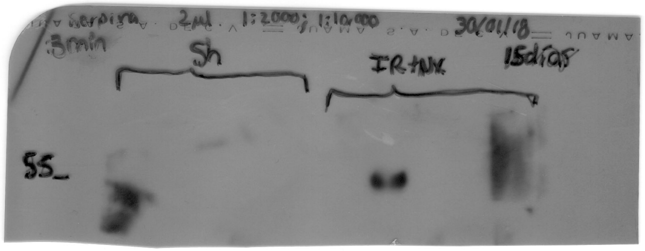

3min

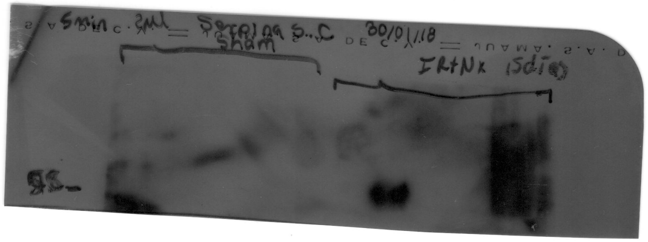

5min

Supplemental Figure 4A

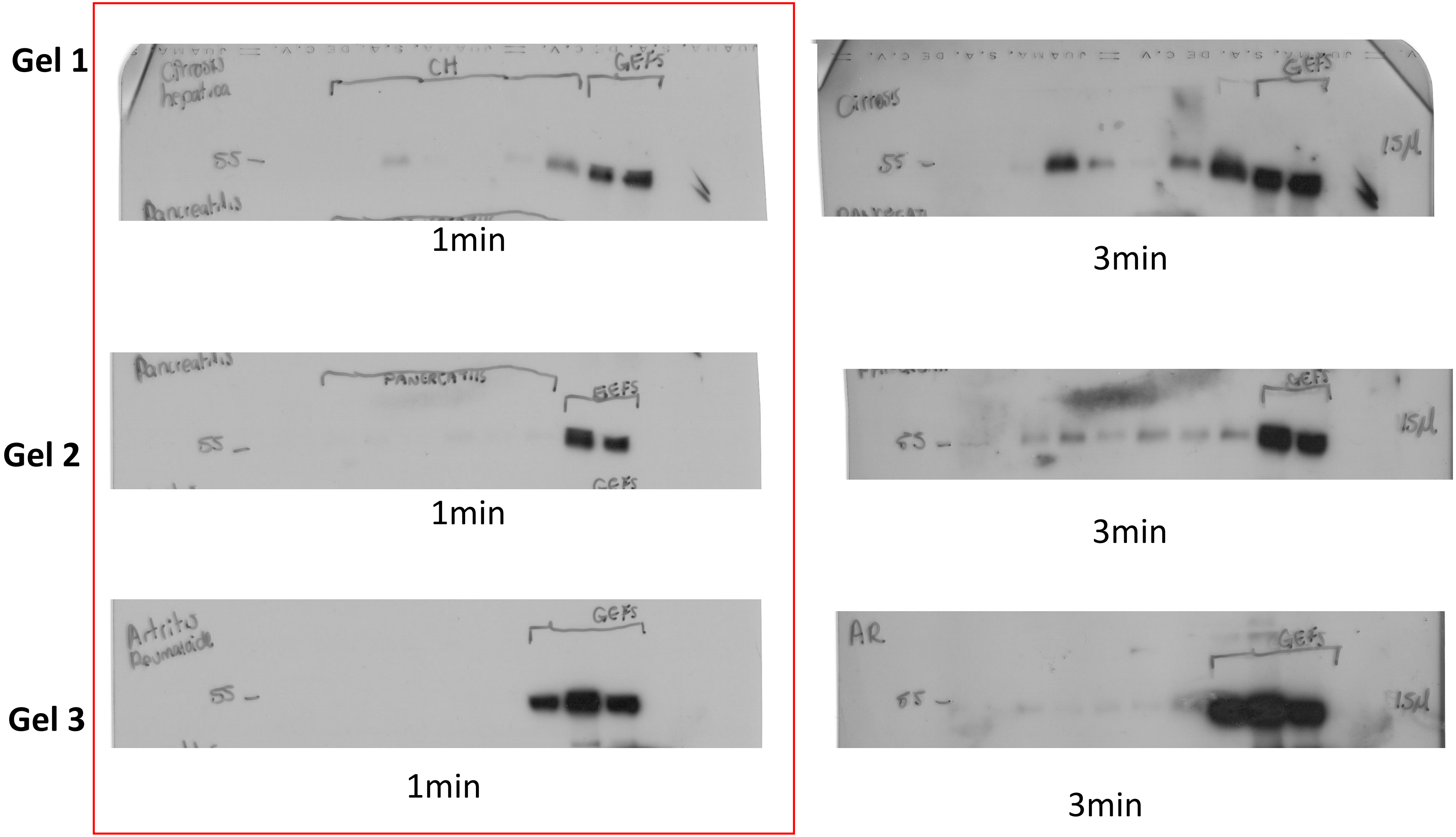

Supplement: Supplementary file 2 — Supplemental Western Blots [file 41598_2019_46601_MOESM2_ESM.pdf]
